# Genome-wide fitness profiling reveals molecular mechanisms that bacteria use to interact with *Trichoderma atroviride* exometabolites

**DOI:** 10.1371/journal.pgen.1010909

**Published:** 2023-08-31

**Authors:** José Manuel Villalobos-Escobedo, Maria Belen Mercado-Esquivias, Catharine Adams, W. Berkeley Kauffman, Rex R. Malmstrom, Adam M. Deutschbauer, N. Louise Glass

**Affiliations:** 1 Plant and Microbial Biology Department, The University of California, Berkeley, California, United States of America; 2 Environmental Genomics and Systems Biology Division, Lawrence Berkeley National Laboratory, Berkeley, California, United States of America; 3 U.S. Department of Energy Joint Genome Institute, Lawrence Berkeley National Laboratory, Berkeley, California, United States of America; GERMANY

## Abstract

*Trichoderma* spp. are ubiquitous rhizosphere fungi capable of producing several classes of secondary metabolites that can modify the dynamics of the plant-associated microbiome. However, the bacterial-fungal mechanisms that mediate these interactions have not been fully characterized. Here, a random barcode transposon-site sequencing (RB-TnSeq) approach was employed to identify bacterial genes important for fitness in the presence of *Trichoderma atroviride* exudates. We selected three rhizosphere bacteria with RB-TnSeq mutant libraries that can promote plant growth: the nitrogen fixers *Klebsiella michiganensis* M5aI and *Herbaspirillum seropedicae* SmR1, and *Pseudomonas simiae* WCS417. As a non-rhizosphere species, *Pseudomonas putida* KT2440 was also included. From the RB-TnSeq data, nitrogen-fixing bacteria competed mainly for iron and required the siderophore transport system TonB/ExbB for optimal fitness in the presence of *T*. *atroviride* exudates. In contrast, *P*. *simiae* and *P*. *putida* were highly dependent on mechanisms associated with membrane lipid modification that are required for resistance to cationic antimicrobial peptides (CAMPs). A mutant in the Hog1-MAP kinase (Δ*tmk*3) gene of *T*. *atroviride* showed altered expression patterns of many nonribosomal peptide synthetase (NRPS) biosynthetic gene clusters with potential antibiotic activity. In contrast to exudates from wild-type *T*. *atroviride*, bacterial mutants containing lesions in genes associated with resistance to antibiotics did not show fitness defects when RB-TnSeq libraries were exposed to exudates from the Δ*tmk3* mutant. Unexpectedly, exudates from wild-type *T*. *atroviride* and the Δ*tmk*3 mutant rescued purine auxotrophic mutants of *H*. *seropedicae*, *K*. *michiganensis* and *P*. *simiae*. Metabolomic analysis on exudates from wild-type *T*. *atroviride* and the Δ*tmk*3 mutant showed that both strains excrete purines and complex metabolites; functional Tmk3 is required to produce some of these metabolites. This study highlights the complex interplay between *Trichoderma*-metabolites and soil bacteria, revealing both beneficial and antagonistic effects, and underscoring the intricate and multifaceted nature of this relationship.

## Author summary

The rhizosphere is composed of plant roots and associated microbes, including fungi. Interactions between roots and rhizosphere bacteria have been intensely investigated, but interactions between bacteria and fungi in the rhizosphere are much less understood. Fungi in the rhizosphere, including the root endophyte, *Trichoderma atroviride*, have the capacity to synthesize and secrete a wide array of complex metabolites that may have a role in shaping both plant and microbe interactions. Using libraries of mutants of rhizosphere bacteria, we show that exposure to *T*. *atroviride* exudates results in a reduction in fitness in bacterial mutants carrying lesions in genes associated with antibiotic resistance and iron transport but an increase in fitness in mutants with lesions in purine biosynthesis. These results show that interactions between bacteria and fungi can be either beneficial or inhibitory, and that interactions between fungi and bacteria can display specificity, such that different bacterial species will have different responses to the presence of fungi. This study shows that *T*. *atroviride* exudates have the potential to shape microbial communities and shows the complexity of interactions between fungi and bacteria in the rhizosphere.

## Introduction

Species in the fungal genus *Trichoderma* are ubiquitous and important members of soil communities [1]. *Trichoderma* species can establish competitive relationships in soil by attacking other fungi, including phytopathogenic fungi [2]. *Trichoderma* species can also establish symbiotic relationships with plants, which generates resistance to infections and improves water and nutrient intake [3]; *Trichoderma* species are often used in commercial soil/seed amendments. We previously reported that the association of *Trichoderma atroviride* with plant roots induces changes in the plant’s gene expression and morphology, even without direct contact with the roots [4]. These phenotypic changes are partially due to the large number of secondary metabolites that *T*. *atroviride* secretes into the environment [5]. *T*. *atroviride* can also suppress the growth of other microbes, including other fungi, by the production of secondary metabolites such as epipolythiodioxopiperazines (ETPs), peptaibols, bisvertinolone, butenolides (harzianolide), pyridones (harzianopyridone), azaphilones, koninginins, steroids, anthraquinones, lactones, trichothecenes, and others compounds that work as chemical weapons [6,7]. *T*. *atroviride* is also a mycoparasite and can degrade and invade fungal cell walls using hydrolytic enzymes that cause necrosis of the host fungus [8].

In addition to interacting with plant roots and other fungi, *Trichoderma* species also interact with many species of bacteria in the rhizosphere. For example, 47 bacterial isolates from the rhizosphere were strongly inhibited by compounds secreted by *T*. *virens* and *T*. *harzianum* [9], while exudates from ten different species of *Trichoderma* had a negative effect on the phytopathogenic bacteria *Ralstonia solanacearum* and *Xanthomonas campestris* [6]. Some compounds produced by *Trichoderma* species, such as peptaibols, which are the product of nonribosomal peptide biosynthesis, have antibiotic activity [10,11]. Such compounds may modify the dynamics of the rhizobiome, as recently reported with *T*. *harzianum* in the rhizosphere of black pepper [12].

Although bacteria and fungi interact in the rhizosphere and compete for space and nutrients [13], very little is known about the molecular mechanisms of root-associated bacterial interactions with *Trichoderma* metabolites. Here, we used *T*. *atroviride*, which can establish a positive relationship with plant roots [2,4,14], to investigate bacterial-fungal interactions. We evaluated large mutant bacterial pools generated by random DNA barcoding mutagenesis (RB-TnSeq) [15] to identify genes important for fitness in the presence of *T*. *atroviride* exudates. We tested three different bacterial species capable of colonizing plant roots and promoting growth, *Klebsiella michiganensis* M5aI [16], *Herbaspirillum seropedicae* SmR1 [17] and *Pseudomonas simiae* WCS417 [18]; both *K*. *michiganensis* M5aI and *H*. *seropedicae* SmR1 can fix nitrogen but belong to different taxonomic classes. In addition, we also investigated *P*. *putida* KT2440 [19], since this is a species routinely isolated from the soil, but is not typically associated with the rhizosphere. *P*. *putida* KT2440 also has various mechanisms of resistance to toxic chemicals, potentially making it an excellent ‘biosensor’ for fungal-bacterial interactions [20,21].

This study uncovered several critical mechanisms that govern the interactions between *T*. *atroviride* exudates and bacteria, including competition for nutrients, especially iron, and the ability to modify membrane lipids associated with tolerance to cationic antimicrobial peptides. In addition, we unexpectedly discovered that plant-associated bacteria can utilize purines secreted by *T*. *atroviride*. This investigation also revealed that a mutation in the *T*. *atroviride* Hog1-MAP kinase gene (Δ*tmk*3) reduced expression of biosynthetic gene clusters that produce secondary metabolites. Exposure of bacterial mutant libraries to Δ*tmk*3 exudates revealed that several bacterial mutants in genes associated with tolerance to antibiotics did not show fitness defects. Analysis of the exudate metabolome of wild type (WT) versus the Δ*tmk3* mutant strains showed significant differences. These data support the hypothesis that the Tmk3 pathway plays a role in the biosynthesis of secondary metabolites, including those capable of inhibiting bacterial growth. These findings underscore the intricate nature of fungal-bacterial interactions in the rhizosphere and shed new light on the complex mechanisms that regulate these interactions.

## Results

### Rhizobacteria are inhibited in growth by *T*. *atroviride* exudates

**Fig 1:**
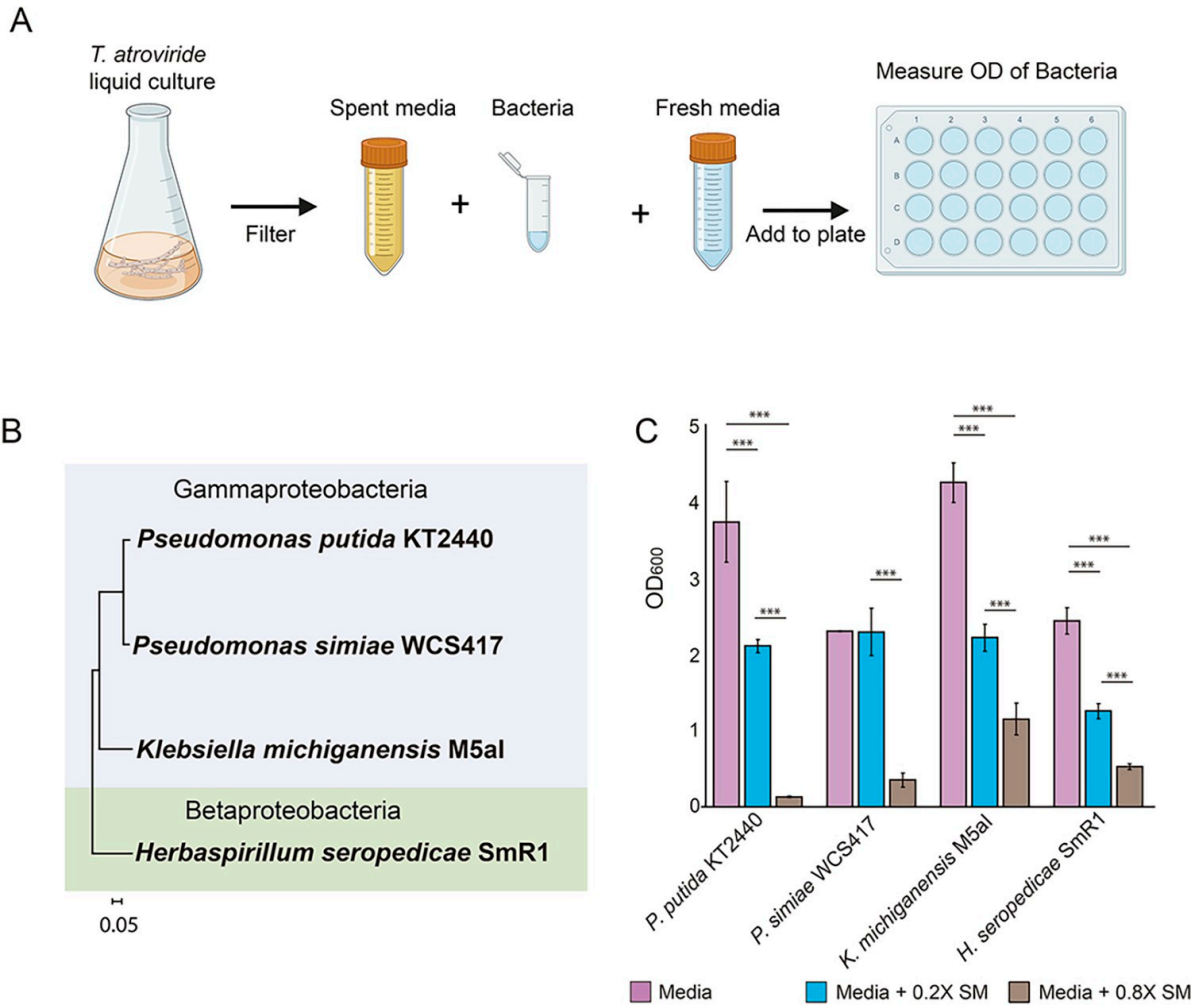
*T*. *atroviride* exudates inhibit the growth of rhizobacteria. **A**) Bacterial cells were incubated for 24 hrs in spent medium (SM) from *T*. *atroviride* cultures (0.2X and 0.8X) with supplementation with fresh medium (R2A at 1X; [66]). The control condition is fresh medium alone (Vogel´s Minimal Media; VMM [64]) supplemented with 1X of R2A. **B)** Phylogenetic distribution of bacteria used in this study using the *rpo*B gene (scale bar 0.05 indicates 5 nucleotide substitutions per 100 nucleotides). **C)** Characterization of the growth of rhizosphere bacteria in response to *T*. *atroviride* exudates (OD600) after 72 hrs at 0.2X and 0.8X concentrations of VMM supplemented with fresh R2A medium. Error bars show SD. Asterisks indicate statistical differences, determined using Tukey’s multiple comparison test (p<0.001; Table B in S1 Text). Fig 1A was constructed via BioRender (https://www.biorender.com), agreement number *BA25LBKUNK*.

We hypothesized that exudates secreted by *T*. *atroviride* would adversely affect growth of rhizosphere bacteria. To test this hypothesis, we evaluated the growth of *P*. *simiae* WCS417, *K*. *michiganensis* M5aI, and *H*. *seropedicae* SmR1 and the soil bacterium *P*. *putida* KT2440 in spent media of *T*. *atroviride* (0.2X and 0.8X) in comparison to growth of these bacteria in uninoculated media (Fig 1 and Tables A and B in S1 Text). As compared to the control condition, all bacterial strains showed growth inhibition in at least one of the two spent media concentrations (Fig 1C). *P*. *simiae* exhibited less susceptibility to *T*. *atroviride* exudates, particularly at the 0.2X concentration (Fig 1C and Table A in S1 Text).

**Fig 2:**
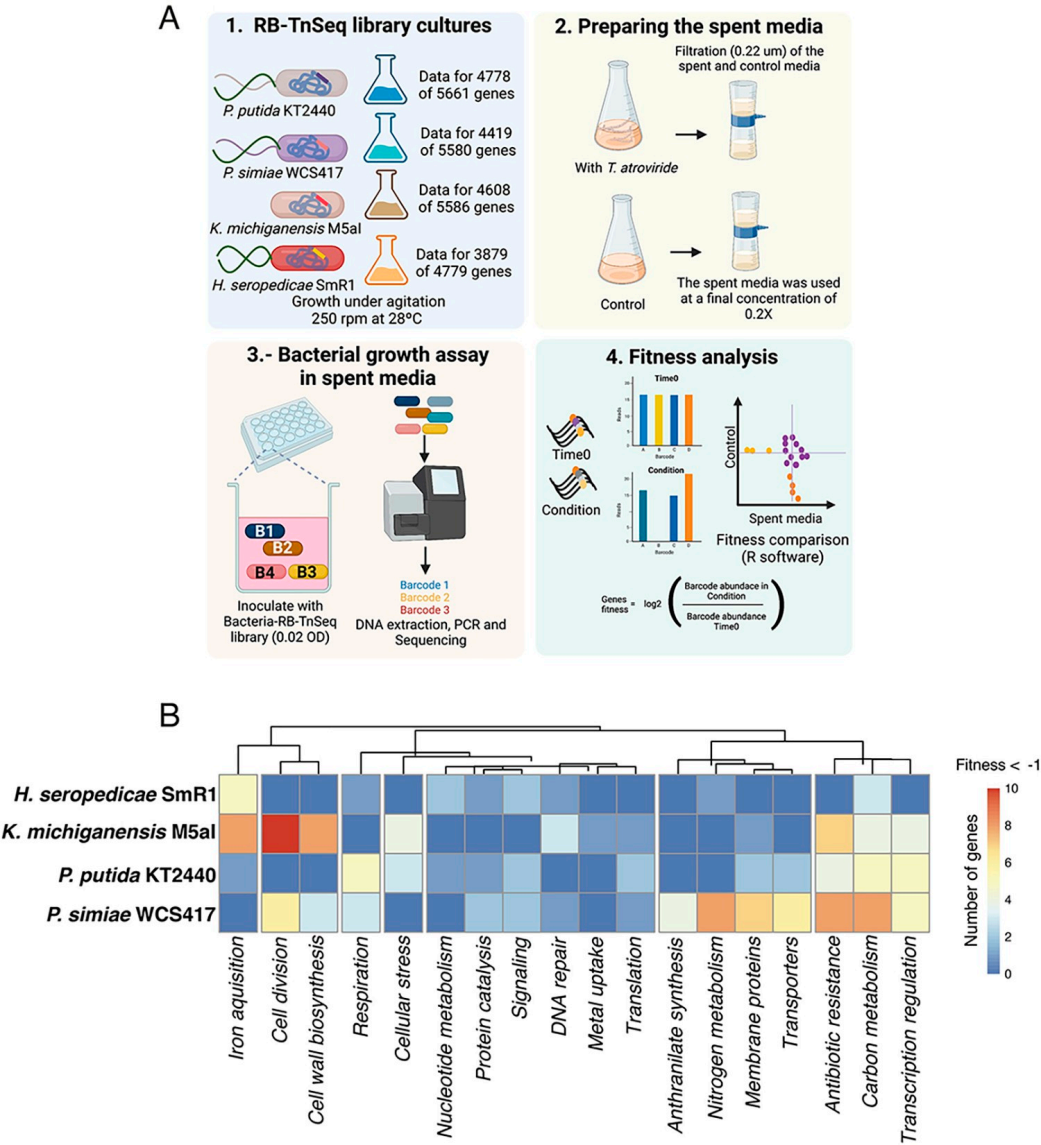
RB-TnSeq of rhizosphere bacteria exposed to *T*. *atroviride* exudates. **A)** Overview of the strategy for RB-TnSeq profiling of rhizosphere bacteria in response to *T*. *atroviride*-spent media. Step 1. RB-TnSeq mutant libraries were grown in LB with kanamycin from archived glycerol stocks. 2. To obtain spent medium, *T*. *atroviride* was grown in Vogel’s Minimal Medium [64] for 72 hrs, then both control and inoculated media were passed through a 0.22 μm filter to remove fungal material. 3. Each bacterial RB-TnSeq library was grown in the presence of *T*. *atroviride* spent media or uninoculated media control. After the bacteria grew for 24 hrs samples were collected for genomic DNA extraction and BarSeq using primers to conserved sequences that flank the unique barcodes. 4. Gene fitness was calculated by comparing the barcode counts in each gene before (Time 0) and after growth in the experimental condition (Condition). **B)** For each bacterium, a heatmap of the main functions of genes with negative fitness when exposed to *T*. *atroviride* exudates (Fitness < -1); detailed predicted functions are provided in S1 Dataset and Fig 2A was constructed via BioRender (https://www.biorender.com), agreement number *DV25LBKS04*.

To determine the mechanisms of bacterial response to exudates from *T*. *atroviride*, we used RB-TnSeq libraries [15,22] constructed for these bacteria to test the fitness of uniquely barcoded and mapped genome-wide insertional mutants; the workflow is shown in Fig 2A. The fitness of mutants in the libraries was evaluated by assessing changes in the frequency of unique barcodes associated with insertions in control versus test conditions. This was achieved by amplifying the unique barcodes using universal primers flanking the barcodes followed by DNA sequencing (BarSeq). If insertions in a particular gene decrease the fitness of mutants under the test conditions, they will show a negative fitness score relative to the control condition. Using a negative fitness filter with a value of -1 in spent media and greater than -1 in control media, we found 17, 60, 41 and 78 genes with negative fitness for *H*. *seropedicae*, *K*. *michiganensis*, *P*. *putida* and *P*. *simiae*, respectively. We classified genes that were affected by *T*. *atroviride* exudates, thereby differentiating 18 distinct functions (Fig 2B and S1 Dataset). Our analysis indicated that *P*. *simiae* and *K*. *michiganensis* depended on a greater diversity of functions associated with exposure to exudates, but that each bacterial species also displayed unique functions as well as some shared processes. For example, in all bacterial species, we observed that genes involved in signaling were important for growth in the presence of fungal exudates.

### Nitrogen-fixing bacteria are strongly affected by iron restriction

**Fig 3:**
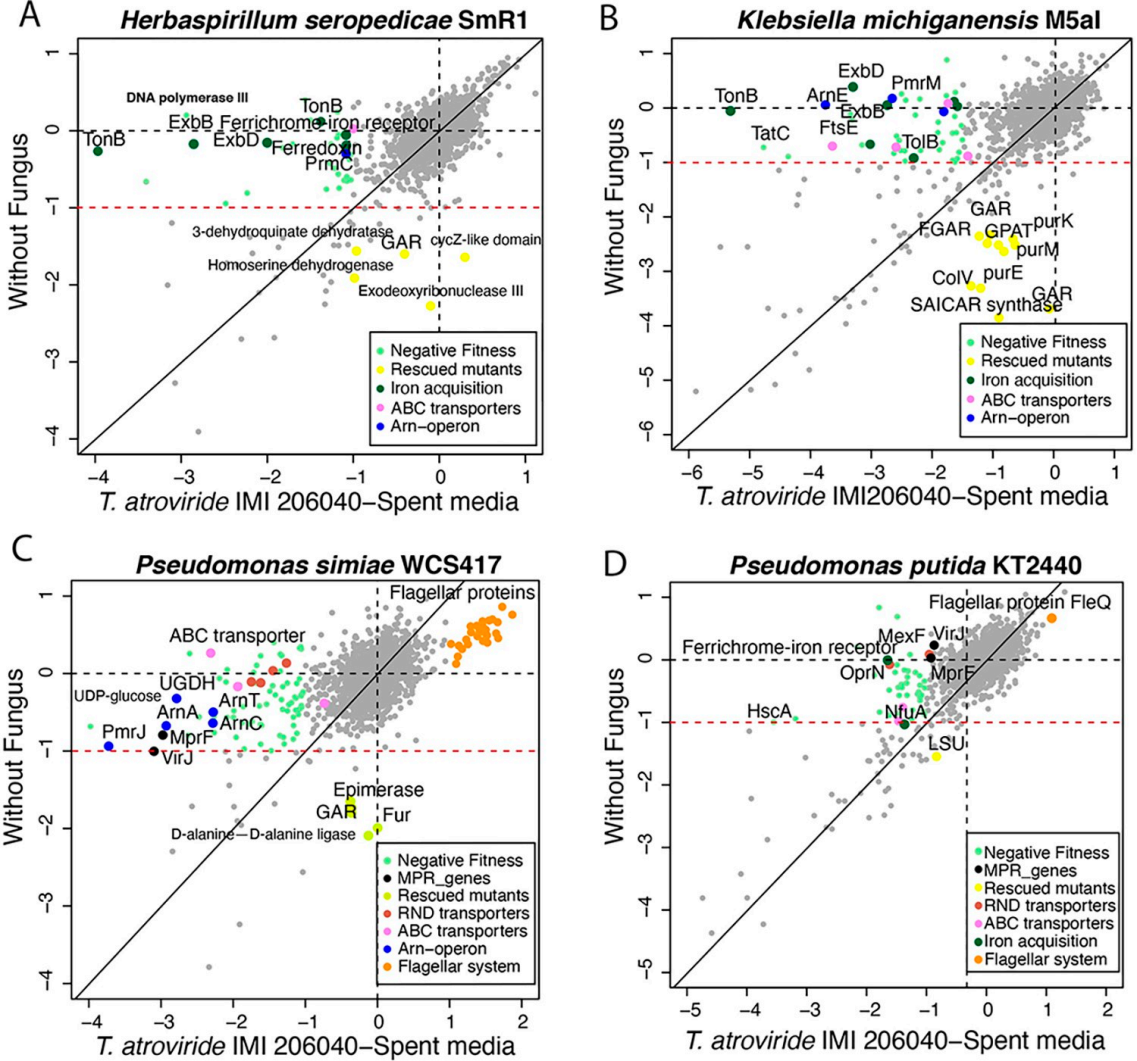
Comparison of bacterial gene log2 fitness in presence and absence of *Trichoderma atroviride*-spent media. At least three replicates per condition were analyzed for all strains. **A)** *Herbaspirillum seropedicae* SmR1, **B)** *Klebsiella michiganensis* M5aI, **C)** *Pseudomonas simiae* WCS417, and **D)** *P*. *putida* KT2440. Each point on the graphs represents the fitness value associated with a disrupted gene, with the gray points indicating no significant change in fitness (|t| > 4). The unnamed genes highlighted in green are those with negative fitness in the presence of *T*. *atroviride* exudates (Fitness < -1 in spent media), while orange points indicate positive fitness values. Shown in yellow are mutants that were phenotypically rescued in the presence of exudates compared to those growing in uninoculated media. Mutations in named individual genes with negative fitness scores in other colors are noted and their general function indicated in the box. Light orange dots represent mutants of the flagellar genes (>1 log2 fitness score).

Fitness assays of the four bacteria showed several genes with significant negative fitness scores (gene fitness < -1 and t < -4, where t is a statistic to measure the significance of an RB-TnSeq gene fitness score [22]) when grown in *T*. *atroviride* exudates relative to uninoculated media (Fig 3). For *H*. *seropedicae* and *K*. *michiganensis*, many of the mutants in genes that showed lower fitness values were related to the TonB-ExbB-ExbD transport system (Fig 3A, 3B and S2 Dataset). The TonB-ExbB-ExbD system is mainly involved in transporting biopolymers such as siderophores that are essential for iron uptake from the environment [23,24,25]. We also detected negative fitness in mutants in a predicted Fe2+/Pb2+ permease. In *T*. *atroviride*, the production of coprogen and ferricrocin, among other siderophores, has been reported [26]. These data suggest that *H*. *seropedicae* and *K*. *michiganensis* faced greater iron limitation when grown in the presence of *T*. *atroviride* exudates, possibly due to the sequestration of iron via fungal siderophores.

In *P*. *putida*, like results with *H*. *seropedicae* and *K*. *michiganensis*, but unlike *P*. *simiae*, disruption of Fe acquisition genes also produced a negative fitness effect. For example, *P*. *putida* mutants in the Ferrichrome-iron receptor and *nfuA* genes (PP_4755 and PP_2378) showed reduced fitness in the presence of *T*. *atroviride* exudates (Fig 3D and S2 Dataset); these genes have previously been shown to be important for acquiring iron [27].

### Antibiotic resistance genes are important for the growth of *P*. *simiae* in the presence of *T*. *atroviride* exudates

Mutants with disruptions in four antibiotic resistance systems in *P*. *simiae* showed reduced fitness when exposed to *T*. *atroviride* exudates (Fig 3C and S2 Dataset). The most affected mutants contained insertions in four Resistance-Nodulation-cell Division (RND)-like genes (PS417_04740, PS417_04745, PS417_17290, and PS417_17295), which function as efflux pumps in bacteria [28]. RND transporters work as a tripartite system located in the inner membrane and participate in detoxification against various antibiotics such as cationic antimicrobial peptides (CAMPs) and dianionic β-lactams [29]; insertions in genes in all three components were important for fitness of *P*. *simiae* in the presence of *T*. *atroviride* exudates. We also observed that mutants in two genes encoding ABC family transporter proteins (PS417_04380 and PS417_24530) also showed reduced fitness when disrupted. These data indicate that one of the main processes triggered in *P*. *simiae* in response to *T*. *atroviride* exudates is to use efflux pumps to remove toxic metabolites from the cell.

Mutants in two Lipid-Modifying Multiple Peptide Resistance genes (*mprF*: PS417_22845 and *virJ* component: PS417_22850) and several genes of the *arnACDEFT* operon also showed reduced fitness when the *P*. *simiae* insertional library was exposed to *T*. *atroviride* exudates (Fig 3C). The *arnACDEFT* operon confers resistance to polymyxin B, an antibiotic that affects the structure of the outer membrane of Enterobacteria [30,31]. Modifying lipids in the cell wall confers resistance to polymyxin B, such as modifications of lipid A with phosphoethanolamine and 4-amino-4-deoxy-L-arabinose.

The results obtained in *P*. *simiae* led us to hypothesize that bacteria in the *Pseudomonas* genus are mainly affected by antibiotics with polymyxin B-like structure and/or mode-of-action in exudates from *T*. *atroviride*. To test this hypothesis, we evaluated the BarSeq profile of the *P*. *putida* RB-TnSeq mutant library exposed to *T*. *atroviride* exudates (Fig 3D and S2 Dataset). In contrast to our hypothesis, we did not observe reduced fitness in mutants of the *arnACDEFT* operon in *P*. *putida*. Indeed, a search for orthologs of these genes in the *P*. *putida* genome showed that at least 7 of the 11 genes for resistance to polymyxin B were absent (Fig A in S1 Text). However, similar to *P*. *simiae*, in *P*. *putida* a negative fitness value was observed in mutants containing insertions in the *mprF* and *virJ* genes that are predicted to be involved in resistance to CAMPs (Fig B in S1 Text) as well as mutants in the *mexF* and *oprN* gene (PP_3427 and PP_3426), which constitute a MexEF-OprN multidrug inner membrane transporter (Fig 3D and S2 Dataset).

### Exposure to polymyxin B resulted in similar fitness profiles in *P*. *simiae* as exposure to *T*. *atroviride* exudates

Our data using *T*. *atroviride* spent media showed that, in *P*. *simiae*, mutants in the *arnACDEFT* operon showed reduced fitness (Fig 3C). The *arnACDEFT* operon is associated with resistance to polymyxin B and colistin in *Klebsiella pneumoniae*, *Pseudomonas aeruginosa*, and *Acinetobacter baumannii* [32]. However, *T*. *atroviride* does not have a biosynthetic gene cluster (BGC) annotated to produce polymyxin B. To determine if the reduced fitness in mutants of *P*. *simiae* was due to the presence of antibiotics in *T*. *atroviride* exudates that potentially act like polymyxins, we first exposed wild-type *P*. *simiae* and *P*. *putida* to polymyxin B. Consistent with the hypothesis that the *P*. *simiae arnACDEFT* operon has a role in resistance to polymyxin B, the WT strain of *P*. *simiae* can resist higher concentrations of this antibiotic as compared to *P*. *putida* (Fig C in S1 Text), which lacks a number of genes in this operon.

**Fig 4:**
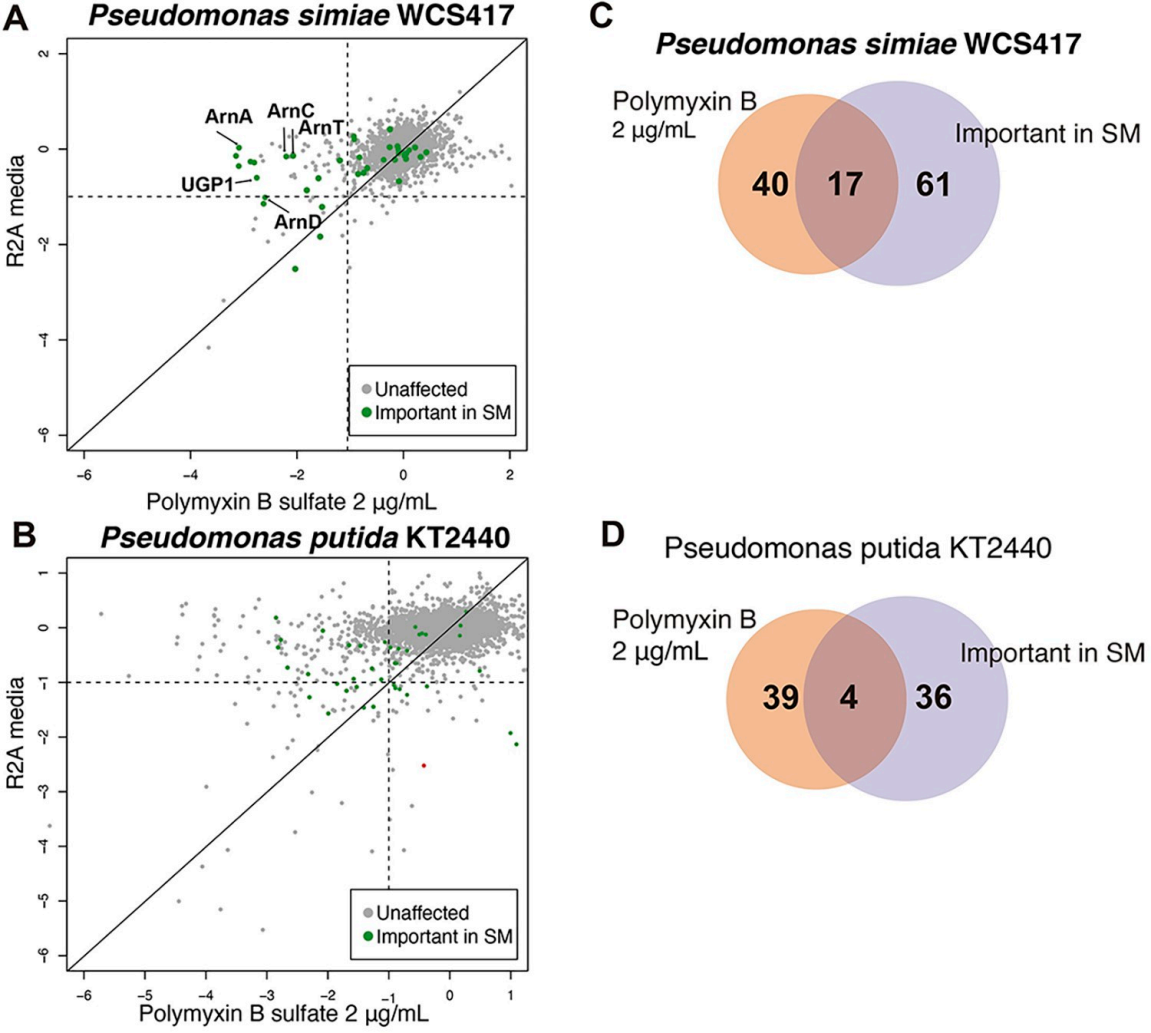
*Pseudomonas simiae* and *P*. *putida* log2 fitness on polymyxin B. At least three replicates per condition were analyzed for all conditions. **A)** BarSeq profile of *P*. *simiae* WCS417 mutant library in response to polymyxin B (2 μg/mL). **B)** BarSeq profile of *P*. *putida* KT2440 mutant library in response to polymyxin B (2 μg/mL). Green dots represent genes with negative fitness in response to *T*. *atroviride* exudates in spent media (SM). **C)** Venn diagram of genes important for fitness in *T*. *atroviride* exudates from SM and genes important for polymyxin B resistance in *P*. *simiae*. **D)** Venn diagram of genes important for fitness on *T*. *atroviride* SM and genes important for polymyxin B resistance in *P*. *putida*. *arnC*: UDP phosphate 4-deoxy-4-formamido-L-arabinose transferase (PS417_13790), *arnA*: UDP-4-amino-4-deoxy-L-arabinose formyltransferase (PS417_13795), *arnT*: 4-amino-4-deoxy-L-arabinose transferase (PS417_13805), *arnD*: 4-deoxy-4-formamido-L-arabinose-phospho-UDP deformylase (PS417_13800), *ugd*: UDP-glucose 6-dehydrogenase (PS417_13820).

Fitness profiling experiments using BarSeq with the RB-TnSeq libraries of *P*. *simiae* in response to polymyxin B (S3 Dataset) revealed that mutants in seventeen of the 78 genes that showed reduced fitness when *P*. *simiae* was exposed to exudates were also affected by exposure to polymyxin B (Fig 4A and 4C; 2 μg/mL), including mutants in all the genes in the *arnACDEFT* operon. This effect was even more striking when 3 μg/mL of polymyxin B was used (Fig D in S1 Text). In contrast, BarSeq experiments with *P*. *putida* revealed that fitness defects in mutants predicted to be involved in antibiotic resistance were not identified in response to polymyxin B exposure (Fig 4B); mutants in only four of the 40 genes affected by *T*. *atroviride* exudates were also affected by exposure to polymyxin B (Fig 4D). These results suggested that the negative impact on the fitness of *P*. *simiae* mutants could be caused by antibiotics that affect the outer membrane, such as polymyxin B. However, attempts to detect polymyxin B by mass spectrometry in *T*. *atroviride* exudates were unsuccessful, suggesting the exudates might contain other compounds with similar chemical structures and/or inhibitory modes of action.

### Validation of RB-TnSeq data using individual transposon mutant strains

**Fig 5:**
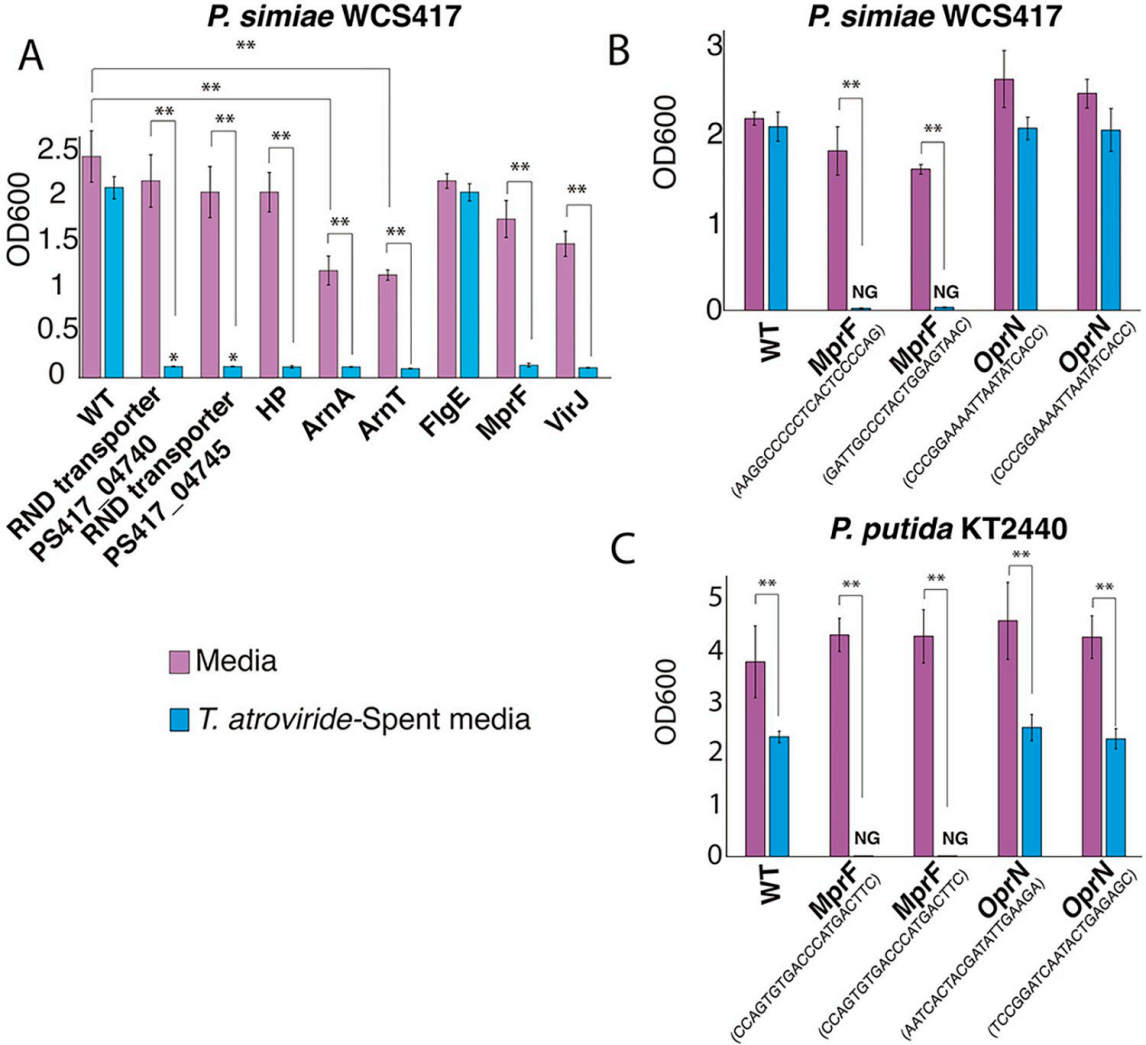
RB-TnSeq validation using single mutants of *P*. *simiae* and *P*. *putida* in response to *T*. *atroviride* exudates. **A)** Eight individual mutants were isolated from the RB-TnSeq library of *P*. *simiae*; the location of insertional mutations were confirmed by sequencing (Table C in S1 Text). Two independent mutants were tested with similar results; one is shown. The profiles correlated with the results obtained from BarSeq fitness profiles. Using two different insertional lines for the *mprF* and *oprN* genes, a growth experiment for 24 hrs was performed with 0.2X spent medium in both *P*. *simiae* (**B**) and *P*. *putida* (**C**). A one-way ANOVA and a Tukey test were performed to determine statistical differences among the different strains in the same treatment (** P < 0.01). Conditions where growth was not observed are labeled NG (no growth).

To validate fitness data indicating that *P*. *simiae* mutants in genes encoding the RND drug efflux pump and mutants in the *arn* operon were sensitive to *T*. *atroviride* exudates, we assayed individual transposon insertion mutants. Insertions in selected genes were verified by Sanger sequencing to confirm the insertion site and the barcode sequence (Table C in S1 Text). We tested two independent mutants in the RND major drug efflux pump (PS417_04740 and PS417_04745) and several independent *P*. *simiae* mutants in *arnA* and *arnT*. All the insertional mutants showed high sensitivity to polymyxin B and were even more negatively affected in growth by exposure to *T*. *atroviride* exudates (Fig 5A).

From BarSeq fitness data, *P*. *simiae* and *P*. *putida* mutants containing insertions in *mprF* (multiple peptide resistance factor) showed reduced fitness in response to *T*. *atroviride* exudates (Fig 3C and 3D and Tables C and D in S1 Text). Furthermore, mutants in *oprN* (efflux pump) showed strong fitness defects in *P*. *putida* as compared to *P*. *simiae*. We therefore tested individual insertional mutant lines in the predicted *mprF* and *oprN* genes in *P*. *simiae* and *P*. *putida* for growth when exposed to *T*. *atroviride* exudates. The growth data showed that the *mprF* mutants in both *P*. *simiae* and *P*. *putida* were highly sensitive to *T*. *atroviride* exudates (Fig 5; P<0.001), indicating that genes involved in resistance to CAMPs were indeed important for fitness. In contrast, *P*. *simiae* strains containing insertions in *oprN* showed a slight, but not significant reduction in growth (Fig 5B), while *P*. *putida* lines containing insertions in *oprN* showed significantly reduced growth (P<0.01) (Fig 5C).

*P*. *simiae* mutants with insertions in flagellar genes showed an increase in fitness when exposed to *T*. *atroviride* exudates in BarSeq-based fitness assays (Fig 3C). However, individual mutants containing insertions in the *flgE* gene, a central gene in the biosynthesis of flagella, did not show increased or reduced growth in response to *T*. *atroviride* exudates (Fig 5A). In other BarSeq experiments (https://fit.genomics.lbl.gov/), mutations that affect motility often led to a fitness increase under laboratory conditions.

### *T*. *atroviride* exudate and fusaric acid fitness profiles do not correlate

One of the genes in the RND system of *P*. *simiae* (PS417_04745) that when mutated showed reduced fitness upon exposure to *T*. *atroviride* exudates is predicted to be a *fuaA* ortholog, which is part of the *fuaR-fuaABC* regulon involved in fusaric acid resistance in the bacterium *Stenotrophomonas maltophilia* [33]. Fusaric acid is a fungal polyketide-derived secondary metabolite first characterized in species within the genus Fusarium and is both phytotoxic and a mycotoxin [34]. Fusaric acid production in *T*. *atroviride* has not been reported, although a biosynthetic gene cluster with 54% similarity to the fusaric acid cluster of *Fusarium verticillioides* [34] was identified in the genome (Fig E in S1 Text). We therefore evaluated the fitness of *P*. *putida* and *P*. *simiae* mutant libraries to pure fusaric acid using BarSeq analysis (Fig F in SI Text and S3 Dataset). However, unlike the polymyxin results, the bacterial response to fusaric acid showed very little correlation with fitness experiments with *T*. *atroviride* exudates (including PS417_04745), even at high concentrations. These results indicated that fusaric acid was absent from *T*. *atroviride* exudates and likely does not play a role in bacterial-fungal interactions under the conditions employed in this study.

### The *T*. *atroviride* Hog1-mitogen activated protein kinase (MAPK) pathway is involved in the biosynthesis of diffusible metabolites that affect bacterial survival

**Fig 6:**
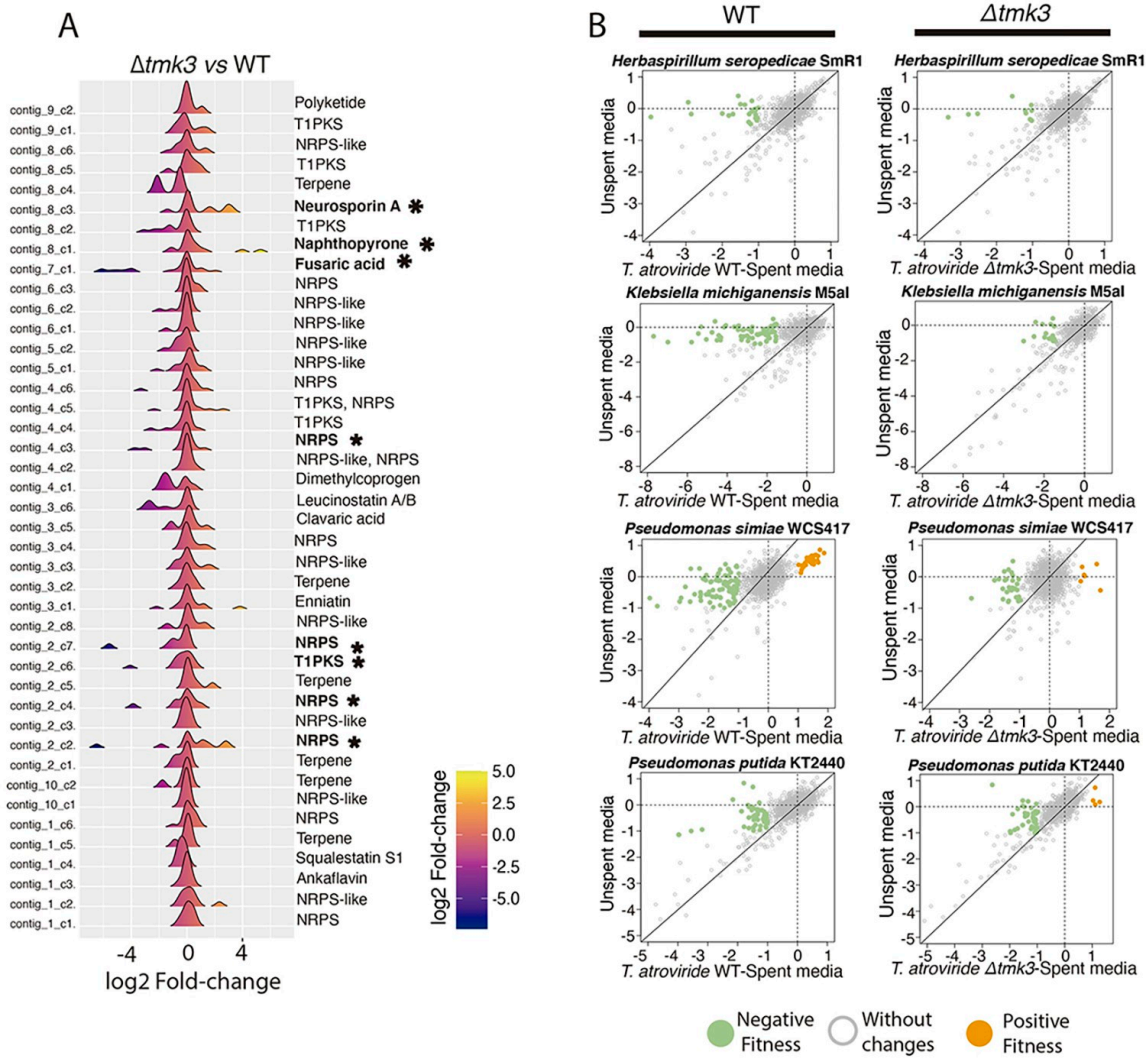
The Hog1-MAPK pathway participates in synthesizing secondary metabolites that affect the fitness of mutants in rhizosphere bacteria. **A**) Analysis of the differential expression of the 42 predicted biosynthetic gene clusters in the Δ*tmk3* mutant *vs*. the WT strain of *T*. *atroviride* (FDR < 0.05). The histograms represent the log2 fold-change values of all the genes within each biosynthetic gene cluster (BGC). At least four Non-Ribosomal Peptide Synthetase (NRPS) gene clusters were affected in their expression in the Δ*tmk3* mutant (mean log2 fold-change < -4). Black asterisks highlight those clusters that on average were repressed or induced more than 4 log2 fold-change. For each BGC, the expression of all the genes within the cluster were evaluated. As the majority of genes within each BGC did not exhibit significant expression changes, the histogram is centered around zero-fold change. **B**) The fitness profile changes in the four microbial strains evaluated when competing against exudates from the Δ*tmk3*-mutant relative to media alone. Green dots represent genes with negative fitness (log2 Fitness < -1 & t-score <-4), and orange dots represent positive fitness (log2 Fitness > 1 & t-score > 4).

We hypothesized that some of the inhibitory compounds in the exudates of *T*. *atroviride* were products of biosynthetic gene clusters BGCs predicted to form secondary metabolites. To test this hypothesis, we first predicted the biosynthetic gene clusters (BGCs) in the genome of *T*. *atroviride* (S4 Dataset); 42 were identified. The Hog1 MAPK Tmk3 regulates signaling cascades and plays an essential role in the regulation of secondary metabolism in *T*. *atroviride* [35,36]. We therefore evaluated differential expression of the 42 predicted BGCs identified in the *T*. *atroviride* genome between the Δ*tmk3* mutant and the wild-type strain using available RNA-seq data (https://www.ncbi.nlm.nih.gov/geo/query/acc.cgi?acc=GSE115811) [37]. We identified 36 repressed genes and 27 induced genes among the 585 genes that constitute the BGCs. The expression of at least four NRPS-like clusters was negatively affected in the Δ*tmk3* mutant, while BGCs that putatively give rise to Neurosporin A and Napthopyrone increased in expression levels (Fig 6A and S4 Dataset). Cationic non-ribosomal peptides and polymyxins are predicted to be synthesized from NRPS biosynthetic clusters, and RNA-seq evidence indicates that expression of at least some of these clusters was affected in the Δ*tmk3* mutant.

We hypothesized that bacterial mutants identified in fitness assays with exudates from *T*. *atroviride* would show a differential response to exudates from the Δ*tmk3* mutant. In support of our hypothesis, a decrease in the number of genes with a significant change in fitness was observed when BarSeq was performed on the four bacterial RB-TnSeq insertional libraries after exposure to Δ*tmk3* mutant exudates (Fig 6B and S2 Dataset). For example, in *P*. *simiae*, insertional mutants in genes encoding efflux pumps and membrane lipid modification genes, antibiotic response genes in the *arnACDEFT* operon and transporters, did not show fitness deficits when exposed to exudates of the Δ*tmk*3 mutant (S2 Dataset). However, in *H*. *seropedicae*, genes involved in iron acquisition were still required for fitness when exposed to exudates from either WT or the Δ*tmk*3 mutant (Fig G in S1 Text). We therefore evaluated the profile of genes involved in iron uptake in conditions that are available in the fitness browser (https://fit.genomics.lbl.gov/cgi-bin/myFrontPage.cgi) (Fig H in S1 Text). This result showed that *H*. *seropedicae*, *K*. *michiganensis* and *P*. *putida* depend on iron uptake genes specifically in the presence of *T*. *atroviride* exudates, regardless of whether it was the WT strain or the Δ*tmk3* mutant. Thus, our data indicate that Tmk3 likely participates in the biosynthesis of secreted metabolites that function as antibiotics but is not involved in the production and release into the environment of siderophore compounds. This inference is also supported by the fact that expression of cluster 4.1, which is predicted to synthesize the dimethylcoprogene siderophore (Fig I in S1 Text), did not change significantly in expression level in the Δ*tmk3* mutant (Fig 6A).

A systematic comparison of bacterial genes important for fitness in exudates from WT *T*. *atroviride* versus the Δ*tmk3* mutant showed that all four bacterial species shared mutations in genes with negative fitness when exposed to either WT or Δ*tmk3* exudates (Fig J in S1 Text). *P*. *simiae* showed the greatest number of unique mutants (61 mutants) affected in fitness upon exposure to WT exudates as compared Δ*tmk3* exudates (10 mutants). In *H*. *seropedicae*, 19 mutants were unique to WT, while only four genes were affected in the Δ*tmk3* strain. In *P*. *putida*, 17 mutants were unique to WT exudates, while 15 were unique to exudates from the Δ*tmk3* strain. Similarly, *K*. *michiganensis* had 29 mutants uniquely affected in fitness when exposed to WT exudates versus 18 unique to Δ*tmk3* exudates.

**Fig 7:**
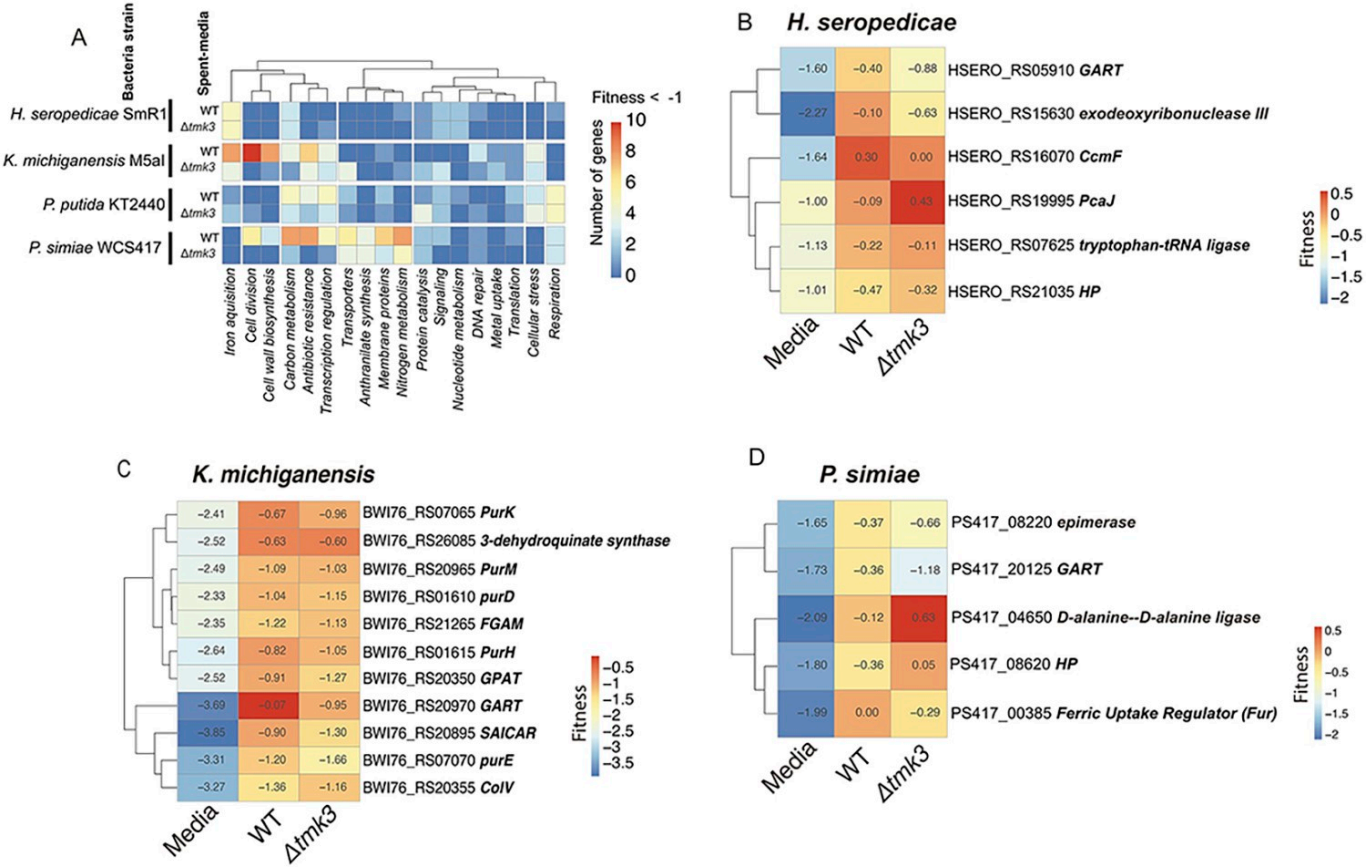
Bacterial processes important for fitness on exudates from WT *T*. *atroviride* and the Δ*tmk3* mutant. **A**) General processes negatively affected in bacterial species by exposure to WT and Δ*tmk3* exudates. The colors of the heatmap represent the number of genes with a significant negative change in fitness (Fitness < -1). **B-D:** Heatmap of gene-fitness values of genes rescued by spent-media of WT and Δ*tmk3* mutant in *H*. *seropedicae* (**B**), *K*. *michiganensis* (**C**), and *P*. *simiae* (**D**) (log2 Fitness in spent media > -1 & log2 Fitness in media < -1.5).

In *K*. *michiganensis*, *P*. *simiae*, and *P*. *putida*, classifying the different bacterial gene mutant functions with negative fitness upon exposure to WT exudates revealed that exudates from the Δ*tmk3* mutant had a reduced impact on fitness of mutants associated with various cellular processes, including cell division, membrane proteins, nitrogen metabolism, and antibiotic resistance (Fig 7A; Table E in S1 Text). Among the different bacterial strains studied, *H*. *seropedicae* displayed the lowest diversity of affected processes, with the primary predicted resistance mechanism to exudates associated with iron transport. In contrast, gene mutants associated with cell division and resistance to antibiotics were no longer negatively affected in fitness in *P*. *simiae* and *K*. *michiganensis* when grown in the presence of Δ*tmk3* mutant exudates.

### The fitness defects of mutants involved in purine synthesis are recovered in *T*. *atroviride* exudates

In addition to fitness deficits in bacterial mutants associated with exposure to T. *atroviride* exudates, mutants in some genes that showed fitness deficits in minimal media recovered after the addition of exudates. For example, *H*. *seropedicae*, *K*. *michiganensis* and *P*. *simiae* mutants with disruptions in purine biosynthesis genes had fitness defects in minimal media, but these growth defects were mitigated when the media was supplemented with *T*. *atroviride* exudates (Fig 7B, 7C and 7D), including glycinamide ribonucleotide transformylase (GART) gene orthologs. GART catalyzes the third step in *de novo* purine biosynthesis, via the transfer of a formyl group to 5’-phosphoribosylglycinamide [38]. In *K*. *michiganensis*, mutants containing insertions in multiple *pur* genes (*purK*, *purM*, *purH*, *and purE*) were rescued by *T*. *atroviride* exudates. These results suggest that *T*. *atroviride* excretes purines into the environment, which some soil bacteria can use.

### The Δ*tmk3* mutant is impaired in the synthesis of large mass charge molecules and accumulates precursor metabolites

**Fig 8:**
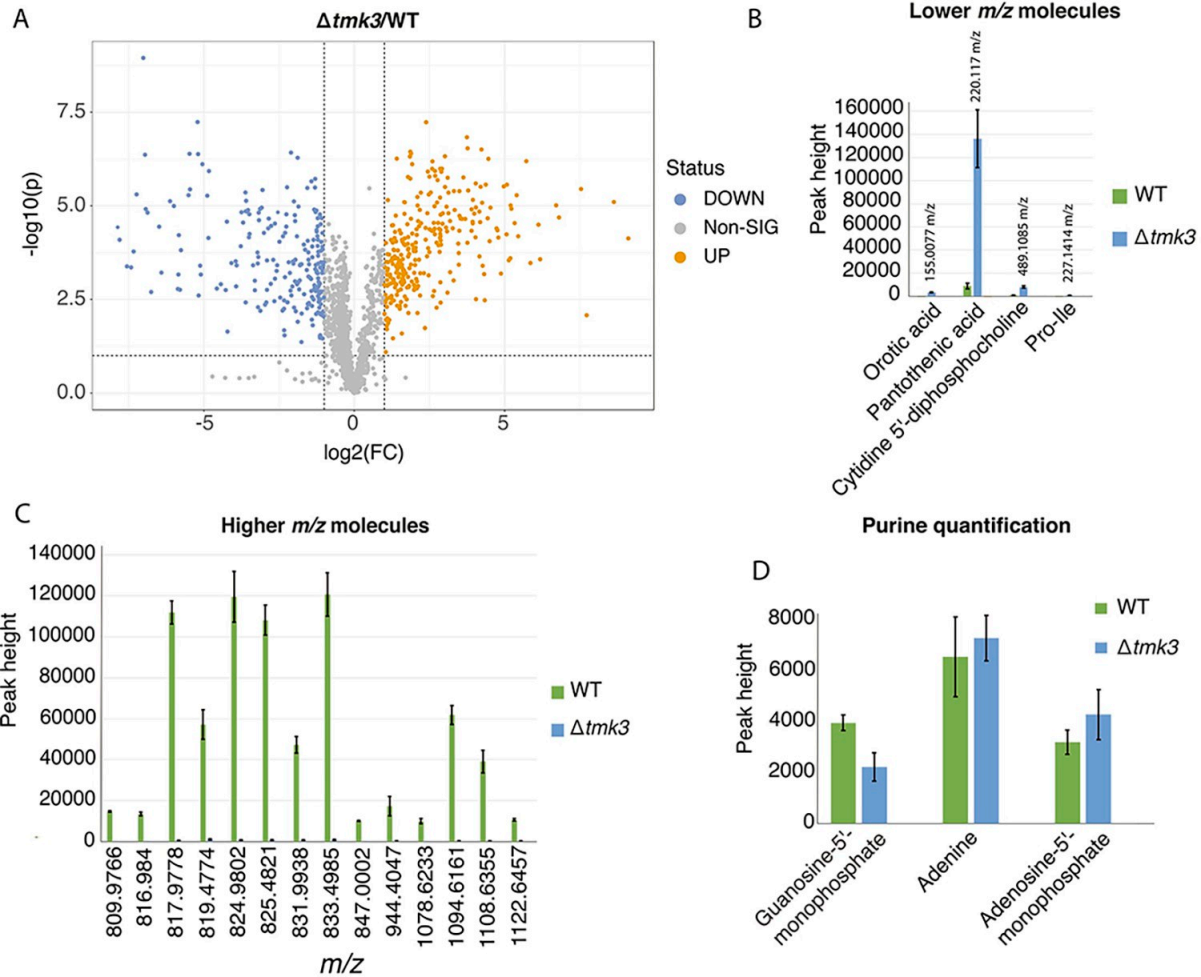
**Hydrophilic Interaction Liquid Chromatography-ESI (Electrospray) QTOF (quadrupole time of flight) MS/MS (tandem mass spectrometry) (HILIC-MS) untargeted metabolomic profile of spent media from wild-type *T*. *atroviride* and the Δ*tmk*3 mutant A**) Volcano plot showing the differential peaks between the Δ*tmk3* mutant versus the WT strain. The x-axis shows the value of log2 fold-change (FC) and the y-axis shows the -log10 value of the adjusted p-value (p). The orange dots show molecules with a log2 fold-change greater than 1, and the blue dots show molecules with log2 fold-change lower than -1 (p-adjusted < 0.05). **B**) Low mass-charge (*m/z*) molecules that are primarily present in the Δ*tmk3* mutant. **C**) Highest *m/z* molecules produced in the WT strain that were greatly reduced in the Δ*tmk3* mutant (Peak height *m/z*). **D**) Purine quantification showed that adenine and adenosine 5´-monophosphate occurred in exudates from *T*. *atroviride* in the same proportion in both WT and the Δ*tmk3* mutant. In bar graphs B, C, and D, the negative controls (media only), were excluded as values were very close to zero (S5 Dataset).

Our RNA-seq analysis and bacterial RB-TnSeq data using exudates from WT *T*. *atroviride* and the Δ*tmk3* mutant indicated that there should be differences in fungal exudate profiles. To test this hypothesis, we performed an untargeted Hydrophilic Interaction Liquid Chromatography-ESI (Electrospray) QTOF (quadrupole time of flight) MS/MS (tandem mass spectrometry) (HILIC-MS) analysis on *T*. *atroviride* exudates (Fig K in S1 Text and S5 Dataset); 1716 different ions were observed, with a mass-charge (*m/z*) range between 57.0344 and 1458.4255. Importantly, the profile of the WT exudates was significantly different from that observed in spent media from the Δ*tmk3* mutant. Statistical analysis to determine the differential ions in Δ*tmk3* relative to WT showed that low *m/z* peaks accumulated in the Δ*tmk3* mutant, while a number of molecules with high *m/z* disappeared (FDR< 0.01; Fig 8). These data suggest that the synthesis of compounds that could have bioactive/antibiotic activities did not accumulate in exudates of the Δ*tmk3* mutant.

The HILIC-MS technique enabled the identification of some of the low *m/z* molecules that were enriched in the Δ*tmk3* exudates (Fig 8B). Among them was pantothenic acid, a precursor of acetyl-CoA, an essential compound in coenzyme A (CoA) synthesis. Acetyl-CoA plays a critical role in lipid synthesis and in the synthesis of NRPS compounds via acyl-CoA ligase and acyl-CoA synthetases [39]. Finally, we confirmed that exudates of both WT and Δ*tmk3* mutant contained purines, including GMP, AMP, and adenine (Fig 8D), supporting our BarSeq data showing that the fitness defects of purine mutants in rhizosphere bacteria were recovered when exposed to exudates from *T*. *atroviride*.

## Discussion

By utilizing RB-TnSeq fitness assays, we systematically identified bacterial genes that are important for fitness in the presence of exudates from *T*. *atroviride*. We identified various gene categories across four bacteria that showed negative fitness when disrupted, including membrane and cell wall maintenance, antibiotic resistance, cellular stress and signaling, iron uptake and metabolism (Fig 2). Thus, RB-TnSeq libraries are a highly versatile tool for evaluating the biological activity of complex exudates and can be utilized as "biosensors", an especially valuable tool when evaluating differences between WT plants/fungi/microbes and selected mutants, as illustrated here using *T*. *atroviride* WT and Δ*tmk3* mutant strains.

Iron is an essential microelement that strongly influences the interactions of microbiota in any environment [40,41,42]. Our findings suggest that *T*. *atroviride* exudates create a highly iron-restrictive environment for bacteria. Consistent with this observation, we predicted at least one BGC synthesizes coprogen (Fig I in S1 Text). Coprogen is a type of siderophore produced by fungi, including *Trichoderma* species, that contains linear dihydroxamate or trihydroxamate moieties [20,43]. In *E*. *coli*, the *fhuE* gene, which belongs to the TonB family, is a promiscuous transporter for coprogen import [43]. Our BarSeq experiments support the hypothesis that *T*. *atroviride* secretes siderophores such as coprogen, and that the nitrogen-fixing bacteria such as *K*. *michiganensis* and *H*. *seropedicae* use the TonB-ExbB-ExbD system to uptake iron in an iron-restrictive environment. Iron deficiency in *H*. *seropedicae* induced genes that encode for a TonB-dependent transporter/transducer, a TonB-dependent receptor, an inner membrane ferrous iron permease [44] and the lipopeptide siderophore, serobactin [45].

Nitrogen-fixing bacteria of the genus *Rhizobium* depend on iron acquisition during symbiosis with legumes [46]. In *Bradyrhizobium japonicum*, TonB2 plays an essential role in the uptake of siderophores from the environment [47]. Recently, the importance of iron in the interaction between fungi and bacteria in cheese rind was demonstrated via BarSeq analysis, which revealed that *E*. *coli* can use siderophores from fungi to obtain iron [48].

Species in the genus *Trichoderma* secrete antibiotics that can inhibit bacterial growth [49], but the question remains whether this is a mechanism of indiscriminate competition or whether these compounds act as a selective force in shaping the microbial composition in the rhizosphere. If the latter is true, it is plausible that this shaping may be due to the presence of different mechanisms of resistance to inhibitory compounds in different bacterial species. One of our key findings was that both *P*. *simiae* and *P*. *putida* rely on multiple antibiotic resistance mechanisms, such as RND and ABC-type efflux pumps to survive exposure to *T*. *atroviride* compounds. This is in line with a recent co-cultivation study using RNA-seq, which investigated the interaction between the rumen bacteria *Fibrobacter succinogenes* and the anaerobic gut fungi *Anaeromyces robustus* or *Caecomyces churrovis* [50]. This study found that while fungi activate different BGCs to produce secondary metabolites, the bacterium *F*. *succinogenes* upregulated five ABC transporter genes and two genes of the efflux RND transporter system, which is predicted to be involved in detoxification under co-culture conditions. These data suggest that bacteria use ABC and RND transporters to remove fungal antibiotic or antibiotic-like compounds from their cytoplasm or periplasm. Our data showed that in *P*. *simiae* and *P*. *putida*, the RND transporter OprN [51] plays a relatively minor role in resistance to *T*. *atroviride* compounds (Fig 5), while MprF was essential in both species.

MprF is a unique enzyme that synthesizes aminoacyl phosphatidylglycerol by modifying phosphatidylglycerol or cardiolipin with distinct aminoacyl groups including lysyl, alanyl, arginyl, and ornithyl groups [52]. This process introduces positive charges on the membrane surface, which decreases the affinity of the membrane for CAMPs. CAMPs are essential components of the eukaryotic immune system to defend against bacterial infections, as they inhibit colonization by bacterial pathogens and aid in the clearance of infections [53]. In the human pathogenic bacterium *Staphylococcus aureus*, MprF is involved in resistance to host antimicrobial peptides [54]. Our results suggest that *T*. *atroviride* potentially secretes a variety of CAMPs into the environment as part of its defense system against bacteria. To our knowledge, this is the first report that the MprF resistance system is required for optimal bacterial growth in the presence of fungal metabolites.

In addition to common mechanisms of resistance to fungal exudates in *P*. *simiae* and *P*. *putida*, *P*. *simiae* possesses unique mechanisms of resistance. In the presence of fungal exudates, *P*. *simiae* showed a strong dependence on the *arnACDEFT* operon, which has been implicated in resistance to polymyxin B [30,31]. This resistance mechanism also consists of modification of lipids in the membrane, specifically lipid A with phosphoethanolamine and 4-amino-4-deoxy-L-arabinose [32]. The presence of both mechanisms for modifying the charge of the cellular membrane in *P*. *simiae* may provide an advantage during interactions with soil fungi, as evidenced by the lower sensitivity of *P*. *simiae* to *T*. *atroviride* exudates (Fig 1C). Previously, the *arnACDEFT* operon in *P*. *simiae* was shown to play an important role during root colonization in *Arabidopsis thaliana* [55], suggesting that modifying bacterial cell membrane charge may help to establish symbiotic relationships between rhizobacteria and plants. In *T*. *atroviride* the production of some of these putative antibiotic compounds are possibly regulated by the Tmk3. It has previously been reported that the Tmk3 signaling pathway is involved in regulating the biosynthesis of some secondary metabolites in both *T*. *atroviride* and *T*. *reesei* [35,56]; our RNA-seq analyses and metabolomics data support these observations.

A third mechanism of interaction was unexpected, as fitness data revealed that plant growth-promoting bacteria can also utilize purines derived from *T*. *atroviride*. In leguminous plants, nodulation defects are observed in purine auxotrophic strains of *Rhizobium fredii* HH303 and *R*. *leguminosarum* 128C56 (bv. Viciae) [57]. However, in *R*. *etli*, purine auxotrophic strains can infect beans if they are supplemented with 5-amino-imidazole-4-carboxamine (AICA) ribose, an intermediate in purine biosynthesis [58]. This evidence suggests that purines may be compounds that some bacteria utilize when produced from other members of the rhizosphere, such as fungi.

One question that remains unanswered is whether purines produced by fungi such as *T*. *atroviride* are primarily used as a nutritional source by rhizosphere bacteria or whether they may also play a signaling role. In *Pseudomonas aeruginosa*, cyclic di-GMP (c-di-GMP) acts as a signal molecule that regulates the change of motility in cells, forming biofilms, which gives the bacteria advantages in resisting different stresses, including resistance to nutrient deprivation or antimicrobial agents [59]. It has also been shown that cAMP and c-di-GMP participate as signaling molecules that activate a series of genes involved in pathogenicity in humans, suggesting these molecules can function in bacteria to activate perception and interaction processes with their hosts [60]. Indeed, something very similar was observed in the colonization of the rhizosphere by *Pseudomonas fluorescens* [61]. Thus, our findings suggest the possibility that purines secreted by *T*. *atroviride* may play a role in promoting proliferation and colonization of certain bacterial species that establish symbiosis with plants in the rhizosphere. In addition to fungi providing nutrients to bacteria, bacteria can also provide needed nutrients to fungi. For example, *Bacillus subtilis* can provide thiamine to hyphae of the filamentous fungus *Aspergillus nidulans*, promoting growth [62], while cooperation between fungi and bacteria has been reported for the degradation of soil organochlorine pesticides such as hexachlorocyclohexane (HCH), where nutrient transfer from soil fungi to bacteria enabled the degradation of HCH by bacteria in contaminated sites [63]. These reports and the data provided in this study highlight the complexities of interactions between fungi and bacteria.

In conclusion, the interaction between rhizosphere bacteria and *T*. *atroviride* exudates is characterized by competition for iron, resistance to antibiotics, and possible antimicrobial peptides produced by the fungal defense system, as well as the possible promotion of growth and colonization through a response to purines. While more research is needed to fully understand how *T*. *atroviride* and the production of its metabolites shape interactions between fungi, bacteria, and plants, our findings suggest that *T*. *atroviride* metabolites can significantly impact microbial interactions in the rhizosphere and alter how microbial populations interact with plants.

## Materials and methods

### *Trichoderma atroviride* and bacterial growth assays and production of spent media

We used the WT strain of *T*. *atroviride* IMI206040 and the isogenic Δ*tmk3* mutant to carry out this work [36]. To obtain fresh conidia, strains were grown in constant light on potato dextrose agar (PDA; Difco) at 28°C for 72 hrs. To obtain spent media, cultures were inoculated with 1x10^6^ conidia per ml in a total volume of 200 ml Vogel’s minimal medium [64] (https://www.fgsc.net/methods/vogels.html) with 2% glucose and incubated for 72 hrs at 28°C with 220 rpm shaking in complete darkness. After 72 hrs, the spent media was separated from the mycelium by two filtration steps with polyethersulfone (PES) filters with a pore size of 0.22 μm. This spent media (SM) was tested for sterility for two days by further incubation to verify there was no additional fungal growth and was stored at 4°C for no more than 48 hrs prior to use in fitness assays.

To calculate the optical density of bacterial cultures, we used a spectrophotometric approach using visible light at a wavelength of 600 nm (OD600). A 1:10 dilution was used to avoid saturation and enable a comparison of growth under control versus test conditions.

### RB-TnSeq *in vitro* growth assays

To carry out the *in vitro* growth assays in this work, we grew previously generated RB-TnSeq libraries from four different species of bacteria: *Klebsiella michiganensis* M5aI, *Herbaspirillum seropedicae* SmR1 [15], *Pseudomonas simiae* WCS417 [18,55] and *P*. *putida* KT2440 [19,65]. One milliliter aliquots of the frozen stock were diluted in 25 mL of LB media supplemented with kanamycin (50 μg/mL). After 24 hrs, the OD600 of the four strains was measured until it reached 1, and 1 ml was centrifuged for each strain. The supernatants were removed, and this time point was used as the control for time 0 (T0). The percentage of genes in the genome that were evaluated for *P*. *putida* KT2440 was 84%, for *P*. *simiae* WCS417 it was 79%, and for *K*. *michiganensis* M5aI and *H*. *seropedicae* SmR1, approximately 82% and 81% of the genes predicted in the genome were evaluated, respectively.

We grew the bacterial RB-TnSeq libraries in *T*. *atroviride* spent media at 0.2X. The spent media was supplemented with 1X R2A [66] fresh medium in a final volume of 1.2 ml (240 μl of R2A at 5X, 240 μl of spent media and 720 μl of H2O). This mix was aliquoted into 24-well plates, and then the four libraries were inoculated to a starting OD600 of 0.02 (from previously grown cell cultures). Three biological replicates were performed for each condition, and uninoculated media was used as controls. Subsequently, the bacterial libraries were cultivated at 28°C for 24 hrs at 250 rpm. All the libraries inoculated on media without *T*. *atroviride* exudates reached ‘culture saturation’ (OD of ~3). Well contents were transferred to an Eppendorf tube and centrifuged at 10,000 rpm for 2 min, and the supernatant was discarded. Mutant library cell pellets were stored at -20°C until genomic DNA extraction.

We extracted the genomic DNA of each RB-TnSeq sample that grew in uninoculated media or in media with the presence of *T*. *atroviride* exudates. Subsequently, PCR was performed to amplify only the barcodes in each sample using universal primers, and amplicons were sequenced on an Illumina HiSeq4000 machine, as described [15,22].

### RB-TnSeq gene fitness calculation

To calculate gene fitness scores from the barcode counts, we used previously gathered information that linked the barcodes in each mutant library to their insertion sites. Gene fitness scores are derived from comparing barcode counts in mutants in that gene from the condition samples to the Time 0 samples (n = 6). The gene fitness is defined as the weighted average of the strain fitness for insertions in the central 10%-90% of the gene. The gene fitness values were normalized such that the typical gene displayed zero fitness. The primary statistic (t) value represents the form of fitness divided by the estimated variance across different mutations of the same gene [22]. In this study, t values of >|4| were considered significant [22,67]. To identify the genes with differential profiles in the presence of spent media, the fitness associated with genes in the control growth condition (media) *vs*. 0.2X spent media, were compared in scatter plots. These genes were evaluated to identify those with positive fitness [Spent Media > 1 & Control < 1]. To identify mutants in genes that showed a recovery phenotype in spent media, the criterion was [Spent Media > -1 & Control < -1.5], and the negative was [Spent Media < -1 & Control >- 1]. For each comparison a threshold of t > 4 was considered. The results of experiments that pass our quality controls are located at the fitness browser, https://fit.genomics.lbl.gov/cgi-bin/myFrontPage.cgi.

### Drug sensitivity assays

To evaluate the RB-TnSeq profile using polymyxin B and fusaric acid, we first performed a growth curve to find the lethal dose for each compound. In the case of polymyxin B, we used concentrations 0, 0.5, 3, 6, and 12 μg/ml diluted in liquid R2A medium. For fusaric acid, concentrations 10, 30, 50, 300, 600 and 900 μg/ml were evaluated. Fusaric acid was dissolved in a 20% methanol solution and added to R2A, with methanol only solutions added to R2A as controls. For quantification, six replicates per condition were performed. 50 mls of bacterial cultures were grown at 250 rpm at 28°C for 24 hrs into 250 ml flasks. Subsequently, the cultures were centrifuged and inoculated into 24-well plates in a total volume of 1.2 ml, with an initial inoculum concentration of 0.02 OD600. For each strain, six uninoculated wells were left as a negative control. Strains were incubated under the same conditions for 24 hrs, and the final OD600 was quantified using the Thermo Scientific AquaMate 7100—Vis Spectrophotometer.

For the RB-TnSeq mutant fitness assays, we evaluated the concentrations of 0.5, 1, 2, 3, and 4 μg/ml for polymyxin B. For fusaric acid, the RB-TnSeq libraries of *P*. *simia*e and *P*. *putida* were profiled with concentrations of 0, 30, 60, 120, 240, and 480 μg/ml. Subsequently, DNA extraction and sequencing were performed to determine the fitness of the mutants of each library in each condition; six replicates were sequenced per evaluated condition.

### Growth test with insertional mutants in single genes

To validate fitness results obtained using *P*. *simiae* and *P*. *putida* RB-TnSeq mutant pools, we performed additional growth assays with individual mutants. Mutants for specific genes were recovered as described [55]. Briefly, individual colonies from mutant pools were transferred from LB + kanamycin (100μg/mL) agar plates into 384-well plates containing LB + kanamycin (100μg/mL) and either 7.5% or 20% glycerol for *P*. *simiae* and *P*. *putida*, respectively. Aliquots from each plate/well were sequenced on an Illumina MiSeq 1x50bp, and the unique RB-TnSeq molecular barcodes were identified for each transposon mutant in each well. At least two mutants with different transposon insertion locations in the target genes were selected for evaluation. Individual mutants were recovered from glycerol stocks, grown on LB + kanamycin agar plates, and verified by Sanger sequencing the RB-TnSeq barcodes of individual colonies.

Growth experiments with individual gene mutants were performed with these strains in 1X R2A medium [66] and 0.2X spent media. The OD600 measurement was performed to determine the growth of each mutant. The growth of the mutants was compared against the growth of the WT strain. These data were graphed with R, and a Tukey test (P<0.05) was applied.

### Identification of biosynthetic gene clusters and differential gene expression analysis

We predicted the biosynthetic gene clusters using antiSMASH [68] with standard parameters in a new version of the *T*. *atroviride* genome sequenced by PacBio (https://www.ncbi.nlm.nih.gov/biosample/SAMN16830915). We determined that there are 42 BGCs encoded in the *T*. *atroviride* genome. Once we identified the genes in each of the predicted BGCs, we associated the expression values with each gene. The FASTQ files were obtained from the Sequence Read Archive (SRA) using the following accession numbers: SRR7343320, SRR7343321, SRR7343322, SRR7343332, SRR7343333, and SRR7343334. After acquiring the data, we performed adapter removal and selected high-quality reads using Trimmomatic Version 0.39. The trimming process involved a sliding window trimming of 4:20; trimmed reads with a minimum length of 36 were retained. High-quality reads were mapped to the *T*. *atroviride* genome (NCBI genome accession number JAEAGS000000000.1) using HISAT2 version 2.1.0 [69], and the read counts were obtained using featureCounts version 2.0.1 [70].

The resulting count table was used to construct an expression matrix for comparing the gene expression profiles of Δ*tmk3-* versus WT-*T*. *atroviride*. To carry out the BGC differential expression analysis between the Δ*tmk3* mutant and the WT strain, we performed the differential expression between both strains to determine the Fold-change value (FDR < 0.05) using edgeR [71,72,73]. The count matrix was normalized using the trimmed mean of M-values (TMM) method [74]. To identify differentially expressed genes, we applied the Generalized Linear Probability Radius Test (GLM) method, setting a Log2 Fold-Change threshold of ±1 and a False Discovery Rate (FDR) of ≤0.05. Data was visualized with ggplot2 in R [75].

### Metabolomic profile of the spent culture media

To elucidate the compounds, present in spent culture media (purines, amino acids, and more complex molecules), we characterized the metabolomic profile of the spent media of *T*. *atroviride* using the service of the West Coast Metabolomics Center at UC Davis (https://metabolomics.ucdavis.edu/). Global analysis to quantify biogenic amines and other small molecule metabolites used HILIC (Hydrophilic Interaction Liquid Chromatography)-ESI (Electrospray) QTOF (quadrupole time of flight) MS/MS (tandem mass spectrometry). Aliquots of 100 μl samples of spent media were resuspended in 80:20 ACN/H2O + iSTD mix. The 1–5 μL samples were injected into an Agilent 1290 UHPLC/Sciex TripleTOF 6600 mass spectrometer equipped with a Waters Acquity Premier UPLC BEH Amide Column (1.7 μm, 2.1 mm x 50 mm). Mobile phase A was Ultrapure water with 10 mM ammonium formate + 0.125% formic acid, pH 3. Mobile phase B was 95:5 v/v acetonitrile:ultrapure water w/ 10 mM ammonium formate + 0.125% formic acid, pH 3. The column temperature was 45°C, while the gradient was 0 min, 100% B; 0.5 min, 100% B; 1.95 min, 70% B; 2.55 min, 30% B; 3.15 min, 100% B; 3.8 min, 100% B. The flow rate 0.8 mL/min.

Data processing chromatograms first underwent a quality control check in which internal standards are examined for peak height and retention time consistency. Raw data files were then processed using an updated version of MS-DIAL software that identifies and aligns peaks and then annotates peaks using an in-house mzRT library and MS/MS spectral matching with NIST/MoNA libraries. All MS/MS annotations were then manually curated by the staff of the WCMC. T-test statistical analysis to determine significant differences between *m/z* peaks was performed using MetaboAnalyst5.0.

## Supporting information

S1 Text **Table A in S1 Text.** Growth measurements (OD600) of *Klebsiella michiganensis* M5aI, *Herbaspirillum seropedicae* SmR1, *Pseudomonas simiae* WCS417 and *Pseudomonas putida* KT2440 in control media (NSP) versus growth in 0.2X and 0.8X in spent media (SM) from *T*. *atroviride-*WT. **Table B in S1 Text.** Statistical tests of bacterial growth in control media versus growth in 0.2X and 0.8X of spent media (SM) from *T*. *atroviride* from three biological replicates. Anova and Tukey’s multiple comparison statistical tests showing significance of growth differences of bacterial species in media or spent media from *T*. *atroviride* (p<0.001). "diff": The difference between the means of two groups being compared. "lwr": The lower bound of the confidence interval for the difference in means. "upr": The upper bound of the confidence interval for the difference in means. "p adj": The adjusted p-value for the comparison between species. Herb = *Herbaspirillum seropedicae* SmR1, *Kleb = Klebsiella michiganensis* M5aI, Put = *Pseudomonas putida* KT2440, Sim = *Pseudomonas simiae* WCS417 and NSP = control media. **Table C in S1 Text**. Individual insertional lines from the random barcoding libraries of *P*. *simiae* WCS417 used in this work. The barcode of each individual mutant was verified by Sanger sequencing. **Table D in S1 Text**. Individual insertional lines from the random barcoding libraries of *P*. *putida* KT2440 used in this work. The barcode of each individual mutant was verified by Sanger sequencing. **Table E in S1 Text.** The number of bacterial genes with negative fitness in the presence of *T*. *atroviride* WT and Δt*mk3* exudates in *H*. *seropedicae*, *K*. *michiganensis*, *P*. *simiae* and *P*. *putida* (Fitness < -1) as identified using BarSeq of the bacterial RB-TnSeq libraries (S2 Dataset). Color scheme is blue (for 0 genes) to white (1 gene) and from pink to red, indicating increased number of genes with negative gene fitness values (S1 Dataset). **Fig A in S1 Text. Schematic representation of the *arnACDEFT* operon that confers resistance to polymyxin B (based on data available for *Pseudomonas aeruginosa and Escherichia coli*** (1, 2). PS417_13790: UDP phosphate 4-deoxy-4-formamido-L-arabinose transferase, *arnC*; PS417_13795: UDP-4-amino-4-deoxy-L-arabinose formyltransferase, *arnA*; PS417_13800: 4-deoxy-4-formamido-L-arabinose-phospho-UDP deformylase *arnD*; PS417_13805: 4-amino-4-deoxy-L-arabinose transferase, *arnT*; PS417_13810: undecaprenyl phosphate-alpha-L-ara4N flippase subunit, *arnE*; PS417_13815: undecaprenyl phosphate-alpha-L-ara4N flippase subunit *arnF*; PS417_13820: UDP-glucose 6-dehydrogenase, *ugd*. Genes that were important for fitness in the four species of bacteria based on BarSeq data (S2 Dataset) when RB-TnSeq mutant libraries were grown in exudates of *T*. *atroviride* are shown in orange. In black are genes that are not represented in the RB-TnSeq mutant library. Blue shows genes that were not important for fitness when grown in *T*. *atroviride* exudates. In violet are genes that do not have an ortholog in *P*. *putida* as compared to *P*. *simiae*, which has the entire *arnACDEFT* operon. **Fig B in S1 Text. Representation of the insertions detected in BarSeq experiments in *P*. *simiae* and *P*. *putida* in the region comprising the MprF system genes (*mprF* and *virJ*).** These genes encode proteins that confer resistance to cationic antimicrobial peptides (CAMPs). The blue dots represent the fitness values of individual transposon mutants with unique DNA barcodes in the corresponding RB-TnSeq mutant library in *P*. *simiae* (3) or *P*. *putida* (4). Green stars indicate *mprF* gene orthologs and yellow stars indicate *virJ* gene orthologs. **Fig C in S1 Text. Bar graph of the effect of polymyxin B sulfate on the growth of *P*. *simiae* WCS417 and *P*. *putida* KT2440**. This experiment was carried out with the WT strains of each bacterium after 24 hrs of growth in R2A or R2A plus polymyxin (5). A one-way ANOVA and a Tukey test were performed to determine statistical differences among the different strains in the same treatment (* p < 0.05; *** p < 0.001). **Fig D in S1 Text.** Effect of mutations in the *arnACDEFT* operon of *P*. *simiae* WSC417 when exposed to 3 μg/ml polymyxin B. Green dots show those mutants that were affected in fitness in the presence of *T*. *atroviride* exudates (genes important for growth on spent media), gray dots indicate those mutants that were not affected by the exudates. **Fig E in S1 Text.** Biosynthetic gene cluster (BGC) of fusaric acid predicted from the *T*. *atroviride* genome (https://www.ncbi.nlm.nih.gov/assembly/GCA_019297715.1**)**. This cluster was classified as number 7.1-type non-ribosomal peptide synthetase (NRPS), T1 polyketide synthase (PKS), NRPS-like. This BGC has 54% similarity to the cluster of *Fusarium verticillioides*, which has been shown to synthesize fusaric acid (6). **Fig F in S1 Text.** RB-TnSeq data on fusaric acid. For *P*. *putida*, concentrations of 120, 240 and 480 ug/ml of fusaric acid were used in the BarSeq experiments (A, B and C); *P*. *putida* can tolerate higher concentrations of this drug. *P*. *simiae* is less tolerant to fusaric acid, so a concentration of 120 ug/ml of fusaric acid was used (D). **Fig G in S1 Text. RB-TnSeq data of *H*. *seropedicae* and *P*. *simiae* in response to exudates from the *T*. *atroviride Δtmk3* mutant.** At least three replicates per condition were analyzed for all strains. A) *Herbaspirillum seropedicae* SmR1 and B) *Pseudomonas simiae* WCS417. The genes not named but are highlighted in green are those with negative fitness in the presence of *T*. *atroviride* exudates (Fitness < -1 in SM), while orange dots indicate positive fitness values. Shown in yellow are mutants that were phenotypically rescued in the presence of exudates as compared to those growing in uninoculated media. Mutations in named individual genes with negative fitness scores in other colors are noted and their general function is indicated in the box. In the panel B, genes belonging to the *arnACDTEF* operon are highlighted in blue, these are circled, since they do not have a change in their fitness significantly unlike what happens in the presence of *T*. *atroviride*-WT exudates. Shown in dark orange are mutants of the RND transporters. Dots in gray represent those mutants that do not have a significant change in their fitness. **Fig H in S1 Text. Gene fitness values for predicted ferrichrome-iron transporters across all growth conditions in the fitness browser (**https://fit.genomics.lbl.gov/cgi-bin/myFrontPage.cgi**).** A) Fitness value comparison of the HSERO_RS00165 and HSERO_RS02660 genes of *H*. *seropedicae* SmR1 in 87 culture conditions (https://fitprivate.genomics.lbl.gov/cgi-bin/myFrontPage.cgi). B) Fitness value comparison of the BWI76_RS05125 and BWI76_RS05130 genes of *K*. *michiganensis* M5aI in 198 culture conditions. Black dots show the fitness of mutants in genes that were affected by growth in *T*. *atroviride* WT exudates. Green dots show the profile in the presence of the Δ*tmk*3 exudates. Blue dots show the fitness of these genes in control medium lacking *T*. *atroviride* exudates (without fungus). Gray dots show the fitness of the genes in the rest of the conditions in the fitness browser. The scale shown on both axes are the gene fitness values. **Fig I in S1 Text. Biosynthetic gene cluster of dimethylcoprogene predicted from the *T*. *atroviride* genome** (https://www.ncbi.nlm.nih.gov/assembly/GCA_019297715.1). This cluster was classified as number 4.1-type non-ribosomal peptide synthetase (NRPS). Dimethylcoprogene may function as a sideophore in *T*. *atroviride* and has been shown to be produced by other filamentous fungi such as *Alternaria alternata* (7). This BGC shows 100% of similarity to the dimethylcoprogen BCG of *A*. *alternata*. **Fig J in S1 Text.** Number of shared and unique genes important for fitness of the four evaluated bacteria exposed to the exudates of WT *T*. *atroviride* and the Δt*mk3* mutant. **Fig K in S1 Text.** Principal Component Analysis (PCA) plot of non-targeted metabolomics profile (S4 Dataset) showing similarities and differences between the three biological replicates (R1, R2 and R3) of the *T*. *atroviride* strain (WT), the *Δtmk3* mutant (Tmk3) and the media control (Ctl). The quality of the representation is the proportion of the total variance of the data that is explained by each of the principal components in the scatterplot. Dimension 1 of the PCA represents 49.6% of the variance in the data, while dimension 2 represents 30.5%.
(DOCX)

S1 Dataset Annotation information for genes that showed negative fitness in *H*. *seropedicae*, *K*. *michiganensis*, *P*. *simiae* and *P*. *putida* onn exposure to *T*. *atroviride* exudates based on biological processes, using GO-terms and published literature. (XLSX)

S2 Dataset RB-TnSeq gene fitness data of *K*. *michiganensis* M5aI, *H*. *seropedicae* SmR1, *P*. *simiae* WCS417 and *P*. *putida* KT2440 from growth in spent and control media from WT *T*. *atroviride* and from the Δ*tmk3* mutant. locus Id: NCBI locus identifier. Description: Annotations from NCBI. Gene fitness scores for replicates 1–3 of each condition.
(XLSX)

S3 Dataset RB-TnSeq gene fitness data of *K*. *michiganensis* M5aI, *H*. *seropedicae* SmR1, *P*. *simiae* WCS417 and *P*. *putida* KT2440 from growth in the presence of different concentrations of the antibiotic polymyxin B or fusaric acid. locus Id: NCBI locus identifier. Description: Annotations from NCBI. Gene fitness scores for replicates 1–3 of each condition.
(XLSX)

S4 Dataset Differential expression of predicted Biosynthetic Gene Clusters (BGCs) identified in 984 the genome of *T*. *atroviride*. Sheet 1: The prediction of the 42 BGCs in the *T*. *atroviride* genome using antiSMASH (45), Sheet 2: Differential expression of all genes in the Δ*tmk3* mutant versus the WT strain using available RNA-seq data (https://www.ncbi.nlm.nih.gov/geo/query/acc.cgi?acc=GSE115811) (39). Sheet 3: The log2FC expression values of each BGCs used for the construction of Fig 6A.
(XLSX)

S5 Dataset Table with the *m/z* of the mass spectrometry data from Vogel’s minimum media (24) (control condition) and spent media from WT *T*. *atroviride* versus the Δ*tmk3* mutant. Fold-change values in the comparison between the Δ*tmk3* mutant and WT per mass spectrometry peak. At the end, the running conditions of this experiment are presented.
(XLSX)
