# Supplementary material for: Genome-wide fitness profiling reveals molecular mechanisms that bacteria use to interact with Trichoderma atroviride exometabolites
Source: PLoS Genet. 2023 Aug 31;19(8):e1010909. doi: 10.1371/journal.pgen.1010909 (PMC10516422; doi:10.1371/journal.pgen.1010909)
Supplement: S1 Text — Table A in S1 Text. Growth measurements (OD600) of Klebsiella michiganensis M5aI, Herbaspirillum seropedicae SmR1, Pseudomonas simiae WCS417 and Pseudomonas putida KT2440 in control media (NSP) versus growth in 0.2X and 0.8X in spent media (SM) from T. atroviride-WT. Table B in S1 Text. Statistical tests of bacterial growth in control media versus growth in 0.2X and 0.8X of spent media (SM) from T. atroviride from three biological replicates. Anova and Tukey’s multiple comparison statistical tests showing significance of growth differences of bacterial species in media or spent media from T. atroviride (p<0.001). "diff": The difference between the means of two groups being compared. "lwr": The lower bound of the confidence interval for the difference in means. "upr": The upper bound of the confidence interval for the difference in means. "p adj": The adjusted p-value for the comparison between species. Herb = Herbaspirillum seropedicae SmR1, Kleb = Klebsiella michiganensis M5aI, Put = Pseudomonas putida KT2440, Sim = Pseudomonas simiae WCS417 and NSP = control media. Table C in S1 Text. Individual insertional lines from the random barcoding libraries of P. simiae WCS417 used in this work. The barcode of each individual mutant was verified by Sanger sequencing. Table D in S1 Text. Individual insertional lines from the random barcoding libraries of P. putida KT2440 used in this work. The barcode of each individual mutant was verified by Sanger sequencing. Table E in S1 Text. The number of bacterial genes with negative fitness in the presence of T. atroviride WT and Δtmk3 exudates in H. seropedicae, K. michiganensis, P. simiae and P. putida (Fitness < -1) as identified using BarSeq of the bacterial RB-TnSeq libraries (S2 Dataset). Color scheme is blue (for 0 genes) to white (1 gene) and from pink to red, indicating increased number of genes with negative gene fitness values (S1 Dataset). Fig A in S1 Text. Schematic representation of the arnACDEFT operon that confers re [file pgen.1010909.s001.docx]

**Genome-wide fitness profiling reveals molecular mechanisms that bacteria use to interact with *Trichoderma atroviride* exometabolites**

José Manuel Villalobos-Escobedo^1,2*^, Maria Belen Mercado-Esquivias^1,2^, Catharine Adams^1,2^, W. Berkeley Kauffman^3^, Rex R. Malmstrom^2,3^, Adam M. Deutschbauer^1,2^, and N. Louise Glass^1,2*^

**Supplementary Tables and Figures**

**Supplementary Tables**

**Table A.** Growth measurements (OD600) of *Klebsiella michiganensis* M5aI, *Herbaspirillum seropedicae* SmR1, *Pseudomonas simiae* WCS417 and *Pseudomonas putida* KT2440 in control media (NSP) versus growth in 0.2X and 0.8X in spent media (SM) from *T. atroviride-*WT.

**Table B.** Statistical tests of bacterial growth in control media versus growth in 0.2X and 0.8X of spent media (SM) from *T. atroviride* from three biological replicates. Anova and Tukey’s multiple comparison statistical tests showing significance of growth differences of bacterial species in media or spent media from *T. atroviride* (p<0.001). "diff": The difference between the means of two groups being compared. "lwr": The lower bound of the confidence interval for the difference in means. "upr": The upper bound of the confidence interval for the difference in means. "p adj": The adjusted p-value for the comparison between species. Herb= *Herbaspirillum seropedicae* SmR1*, Kleb= Klebsiella michiganensis* M5aI, Put= *Pseudomonas putida* KT2440, Sim= *Pseudomonas simiae* WCS417 and NSP=control media.

**Table C.**  Individual insertional lines from the random barcoding libraries of *P. simiae* WCS417 used in this work. The barcode of each individual mutant was verified by Sanger sequencing.

**Table D.**  Individual insertional lines from the random barcoding libraries of *P. putida* KT2440 used in this work. The barcode of each individual mutant was verified by Sanger sequencing.

**Table E.**  The number of bacterial genes with negative fitness in the presence of *T. atroviride* WT and Δt*mk3* exudates in *H. seropedicae*, *K. michiganensis*, *P. simiae* and *P. putida* (Fitness < -1) as identified using BarSeq of the bacterial RB-TnSeq libraries (Supplementary Dataset 2). Color scheme is blue (for 0 genes) to white (1 gene) and from pink to red, indicating increased number of genes with negative gene fitness values (S1 Dataset).

**Supplementary Figures**

**
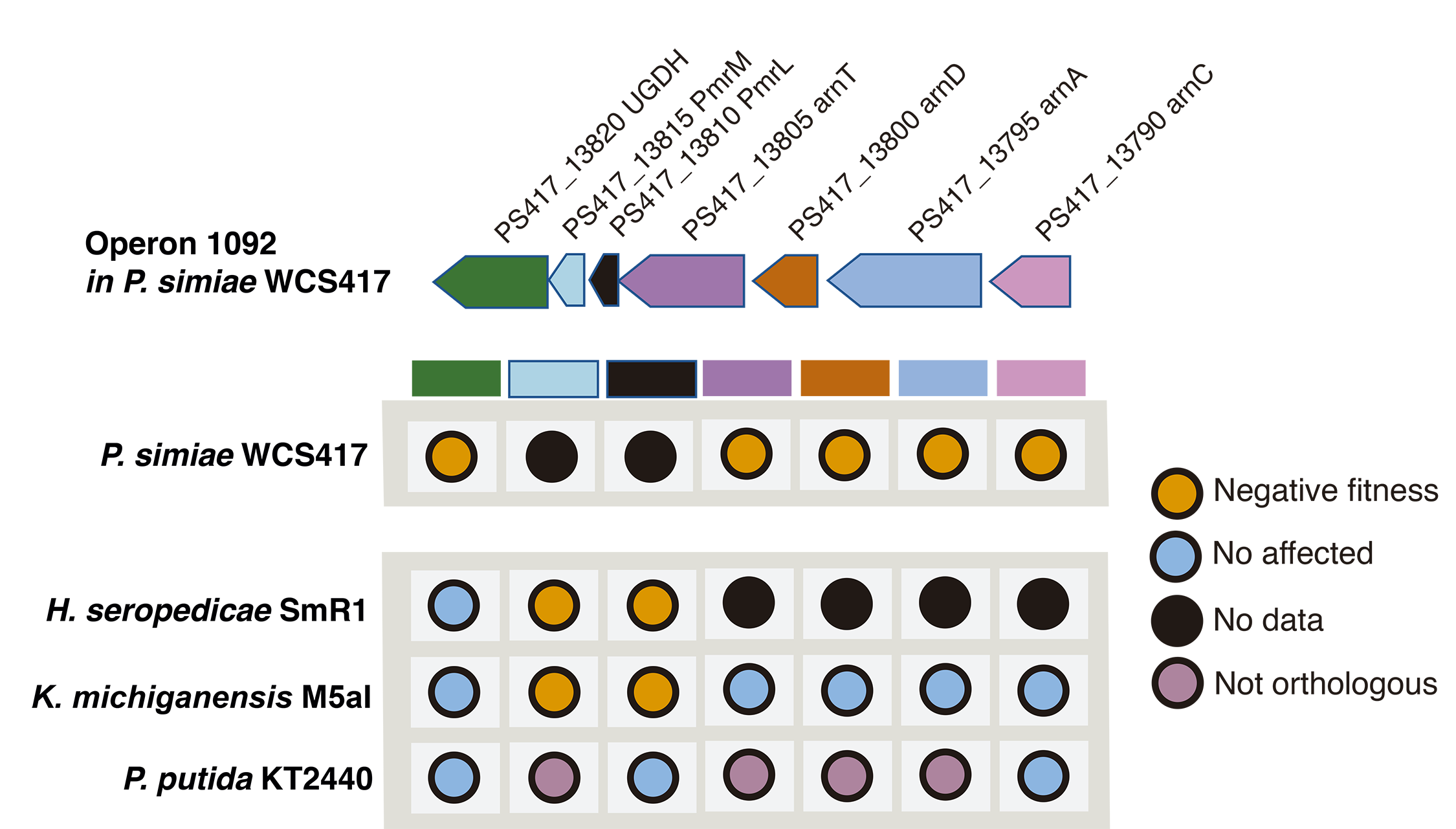
**

**Fig A. Schematic representation of the arnACDEFT operon that confers resistance to polymyxin B (based on data available for *Pseudomonas aeruginosa and Escherichia coli*** (1, 2). PS417_13790: UDP phosphate 4-deoxy-4-formamido-L-arabinose transferase, *arnC*; PS417_13795: UDP-4-amino-4-deoxy-L-arabinose formyltransferase, *arnA*; PS417_13800: 4-deoxy-4-formamido-L-arabinose-phospho-UDP deformylase *arnD*; PS417_13805: 4-amino-4-deoxy-L-arabinose transferase, *arnT*; PS417_13810: undecaprenyl phosphate-alpha-L-ara4N flippase subunit, *arnE*; PS417_13815: undecaprenyl phosphate-alpha-L-ara4N flippase subunit *arnF*; PS417_13820: UDP-glucose 6-dehydrogenase, *ugd*. Genes that were important for fitness in the four species of bacteria based on BarSeq data (Dataset 2) when RB-TnSeq mutant libraries were grown in exudates of *T. atroviride* are shown in orange. In black are genes that are not represented in the RB-TnSeq mutant library. Blue shows genes that were not important for fitness when grown in *T. atroviride* exudates. In violet are genes that do not have an ortholog in *P. putida* as compared to *P. simiae*, which has the entire arnACDEFT operon.


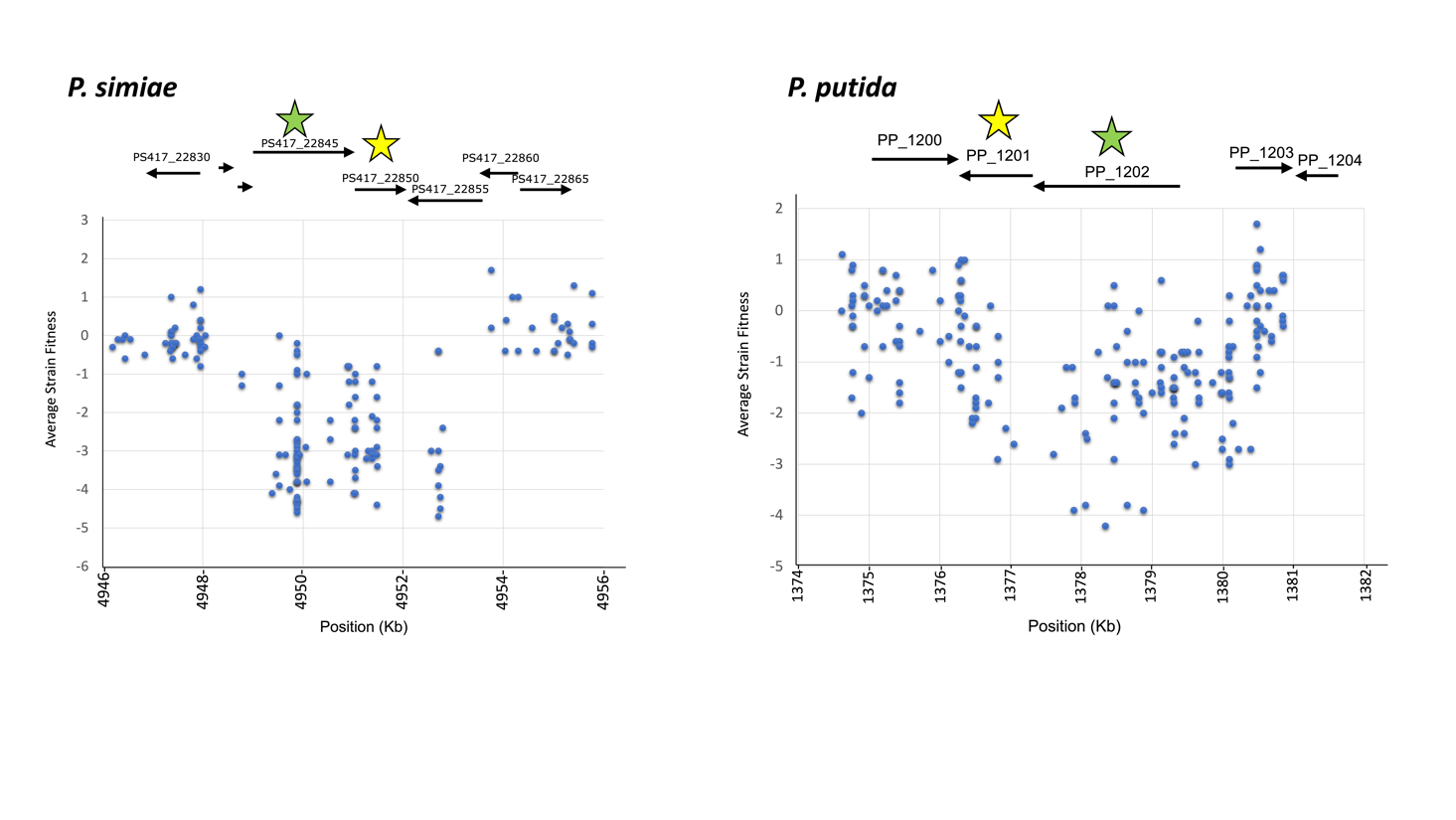


**Fig B. Representation of the insertions detected in BarSeq experiments in *P. simiae* and *P. putida* in the region comprising the MprF system genes (*mprF* and *virJ*).** These genes encode proteins that confer resistance to cationic antimicrobial peptides (CAMPs). The blue dots represent the fitness values of individual transposon mutants with unique DNA barcodes in the corresponding RB-TnSeq mutant library in *P. simiae* (3) or *P. putida* (4). Green stars indicate *mprF* gene orthologs and yellow stars indicate *virJ* gene orthologs.


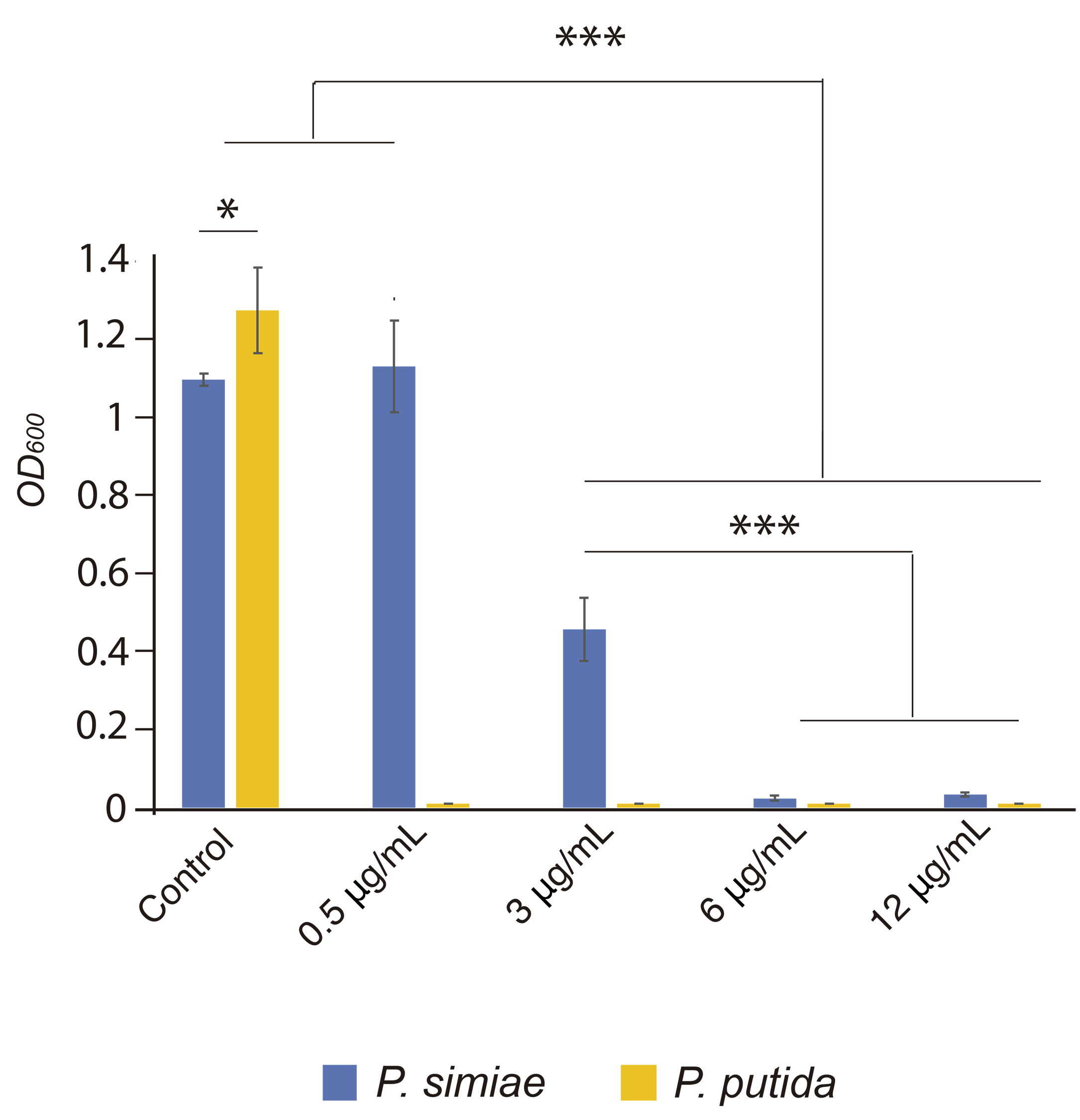


**Fig C.** **Bar graph of the effect of polymyxin B sulfate on the growth of *P. simiae* WCS417 and *P. putida* KT2440** This experiment was carried out with the WT strains of each bacterium after 24 hrs of growth in R2A or R2A plus polymyxin (5). A one-way ANOVA and a Tukey test were performed to determine statistical differences among the different strains in the same treatment (* p < 0.05; *** p < 0.001).

**
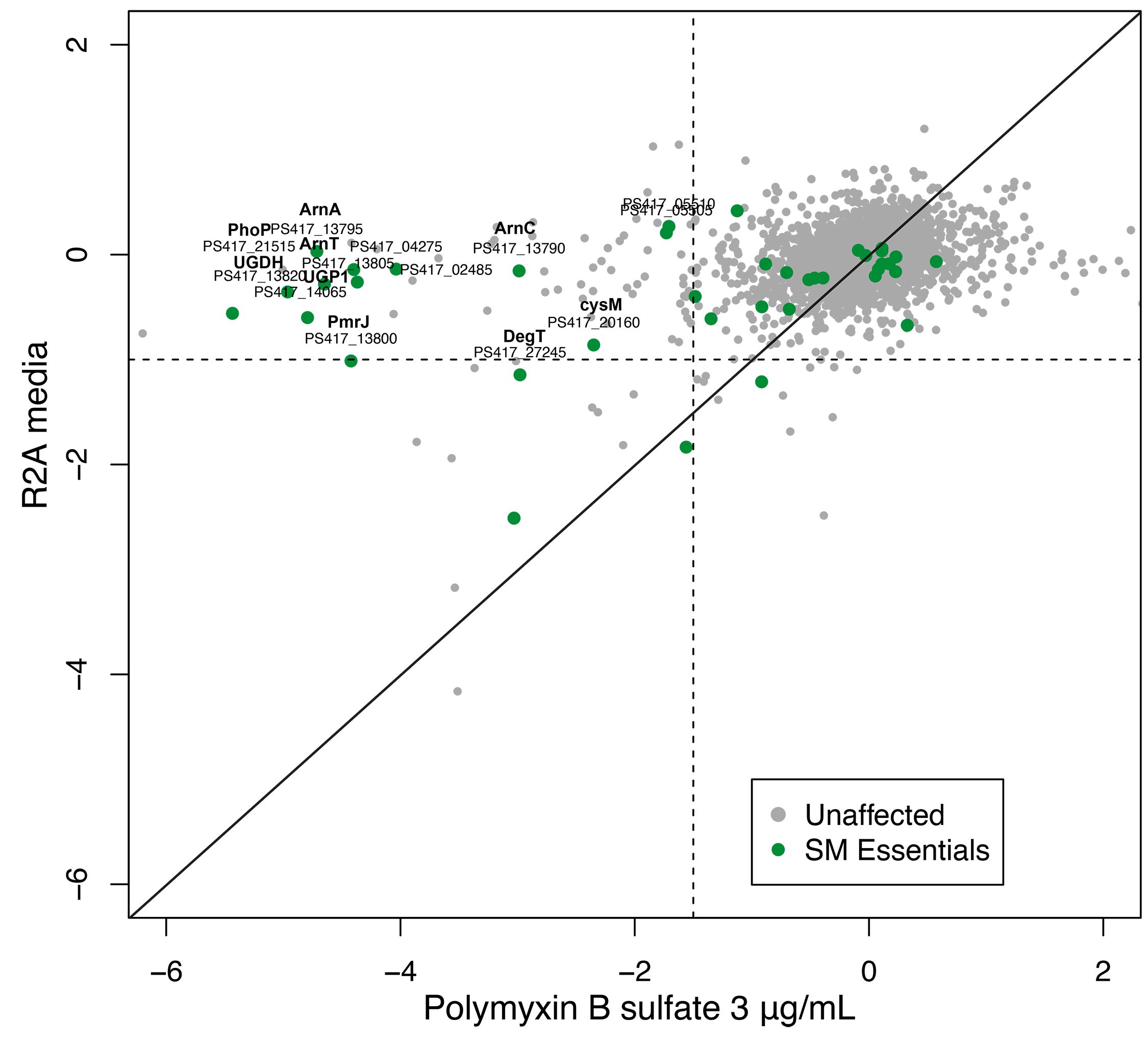
**

**Fig D.** Effect of mutations in the arnACDEFT operon of *P. simiae* WSC417 when exposed to 3 µg/ml polymyxin B. Green dots show those mutants that were affected in fitness in the presence of *T. atroviride* exudates (genes important for growth on spent media), gray dots indicate those mutants that were not affected by the exudates.


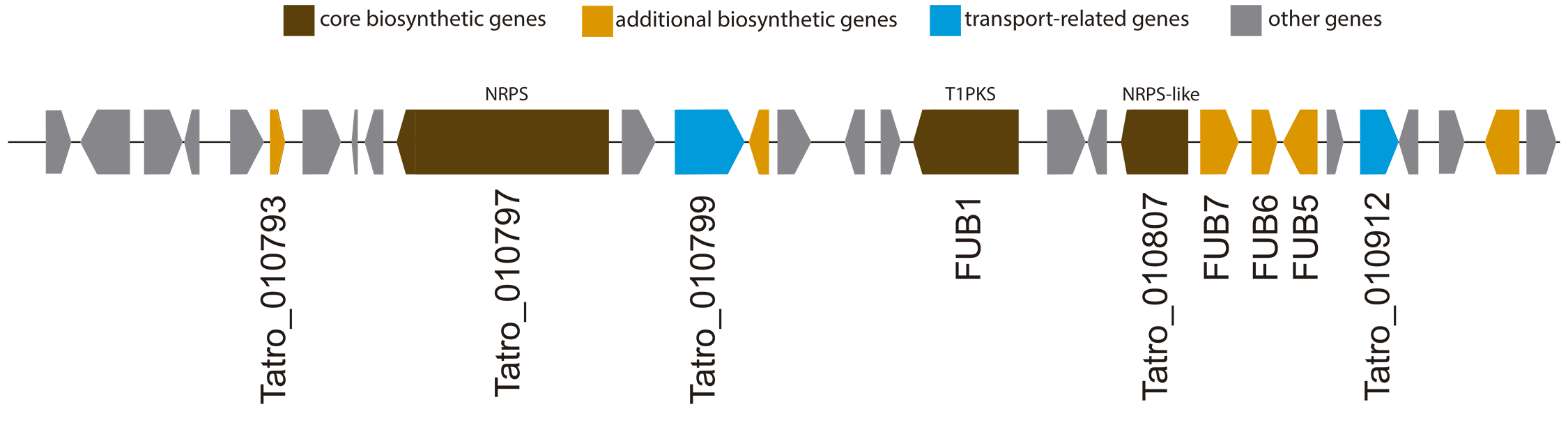


**Fig E.** Biosynthetic gene cluster (BGC) of fusaric acid predicted from the *T. atroviride* genome (<https://www.ncbi.nlm.nih.gov/assembly/GCA_019297715.1>**)**. This cluster was classified as number 7.1-type non-ribosomal peptide synthetase (NRPS), T1 polyketide synthase (PKS), NRPS-like. This BGC has 54% similarity to the cluster of *Fusarium verticillioides,* which has been shown to synthesize fusaric acid (6).


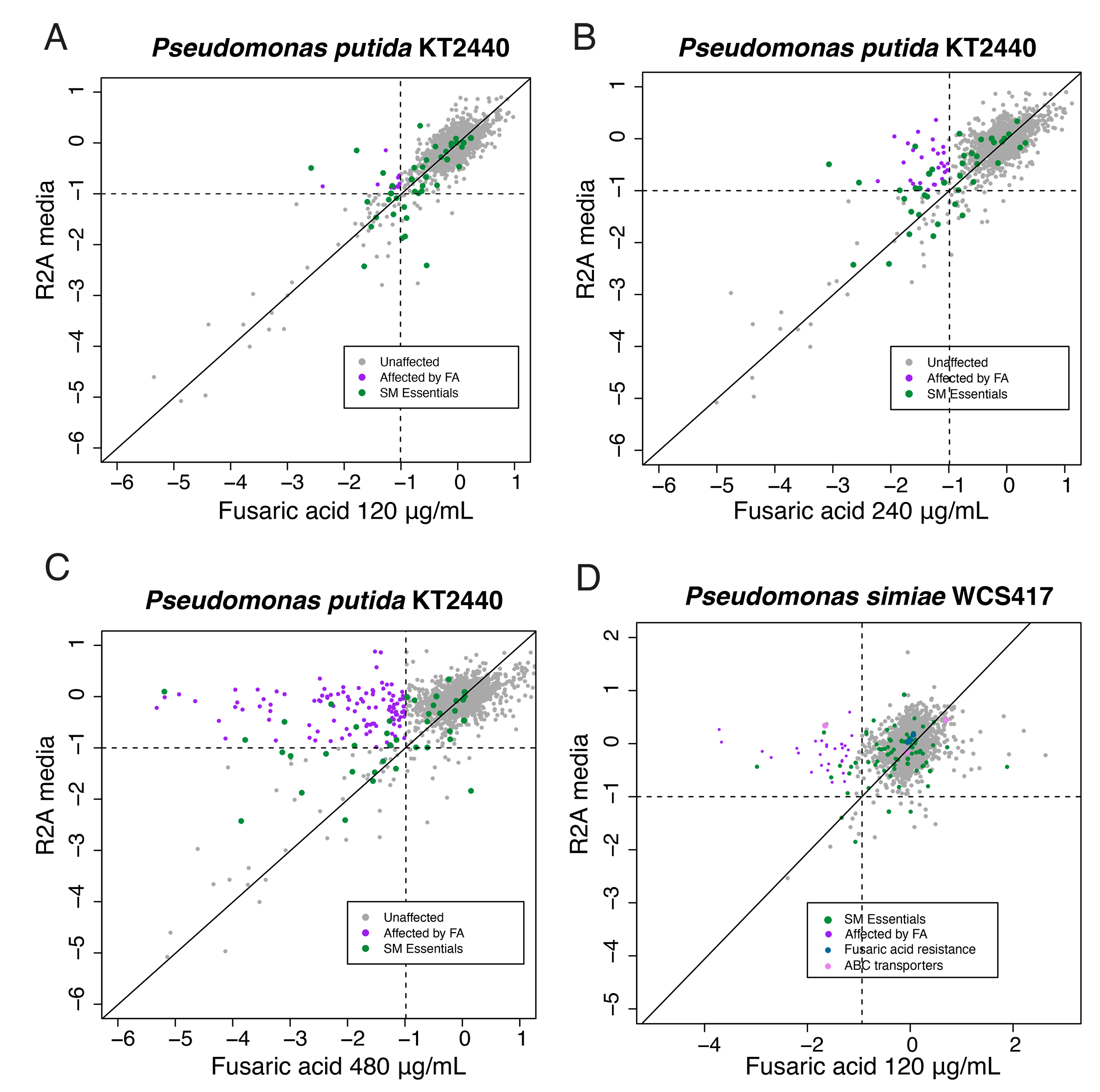


**Fig F.** RB-TnSeq data on fusaric acid. For *P. putida*, concentrations of 120, 240 and 480 ug/ml of fusaric acid were used in the BarSeq experiments (A, B and C); *P. putida* can tolerate higher concentrations of this drug. *P. simiae* is less tolerant to fusaric acid, so a concentration of 120 ug/ml of fusaric acid was used (D).

**
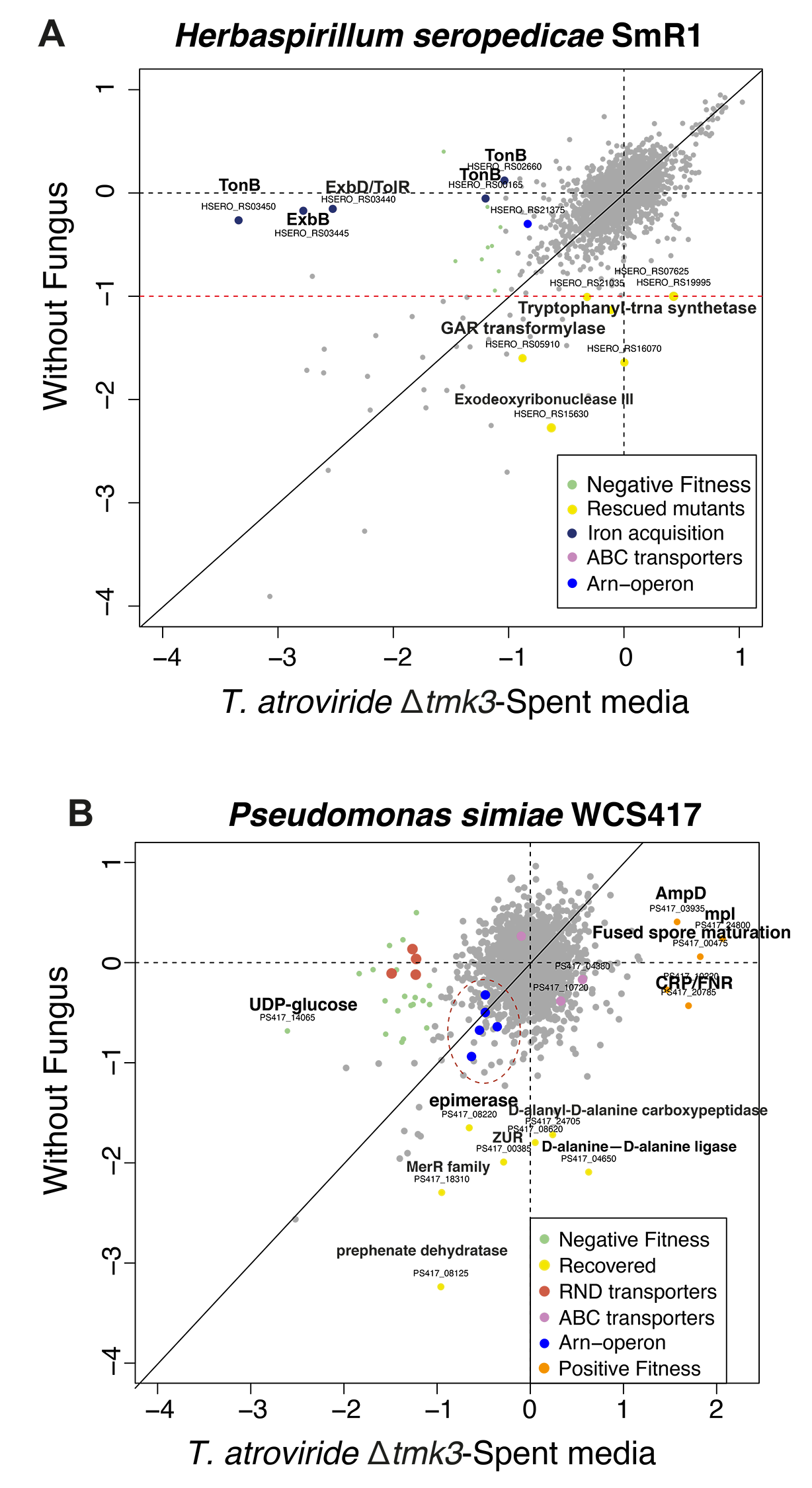
**

**Fig G. RB-TnSeq data of *H. seropedicae* and *P. simiae* in response to exudates from the *T. atroviride* *Δtmk3* mutant.** At least three replicates per condition were analyzed for all strains. A) *Herbaspirillum seropedicae* SmR1 and B) *Pseudomonas simiae* WCS417*.*  The genes not named but are highlighted in green are those with negative fitness in the presence of *T. atroviride* exudates (Fitness < -1 in SM), while orange dots indicate positive fitness values. Shown in yellow are mutants that were phenotypically rescued in the presence of exudates as compared to those growing in uninoculated media. Mutations in named individual genes with negative fitness scores in other colors are noted and their general function is indicated in the box. In the panel B, genes belonging to the *arnACDTEF* operon are highlighted in blue, these are circled, since they do not have a change in their fitness significantly unlike what happens in the presence of *T. atroviride*-WT exudates. Shown in dark orange are mutants of the RND transporters. Dots in gray represent those mutants that do not have a significant change in their fitness.

**
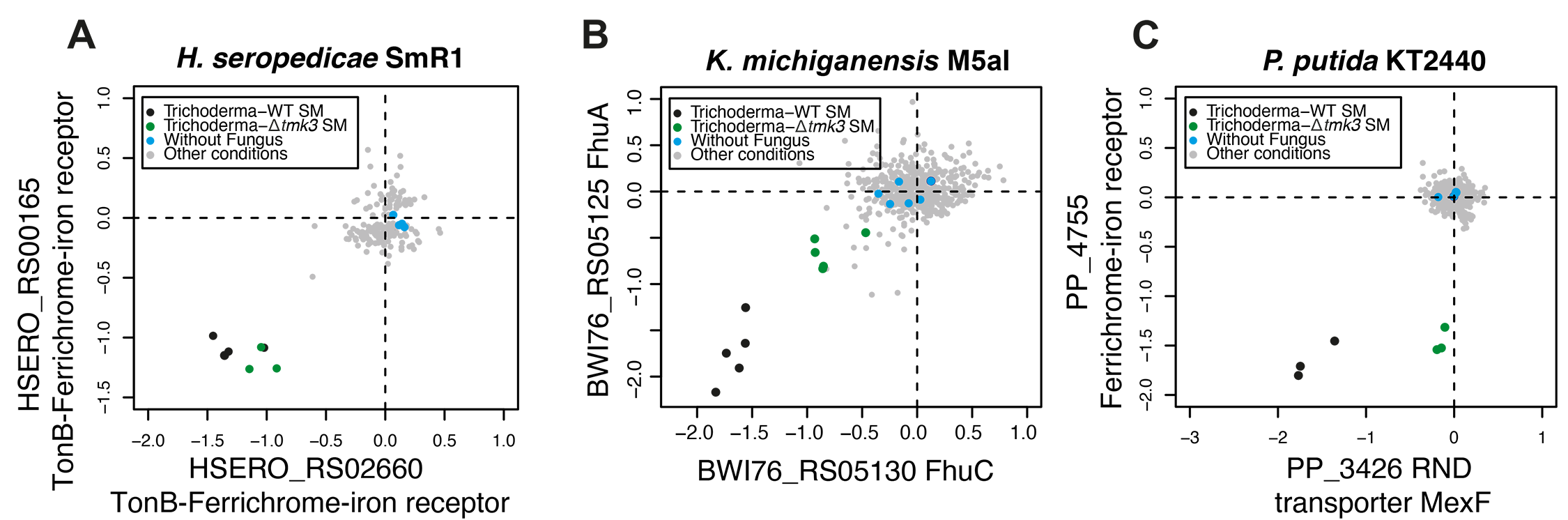
**

**Fig H.** **Gene fitness values for predicted ferrichrome-iron transporters across all growth conditions in the fitness browser (https://fit.genomics.lbl.gov/cgi-bin/myFrontPage.cgi).** A) Fitness value comparison of the HSERO_RS00165 and HSERO_RS02660 genes of *H. seropedicae* SmR1 in 87 culture conditions (https://fitprivate.genomics.lbl.gov/cgi-bin/myFrontPage.cgi). B) Fitness value comparison of the BWI76_RS05125 and BWI76_RS05130 genes of *K. michiganensis* M5aI in 198 culture conditions. Black dots show the fitness of mutants in genes that were affected by growth in *T. atroviride* WT exudates. Green dots show the profile in the presence of the Δ*tmk*3 exudates. Blue dots show the fitness of these genes in control medium lacking *T. atroviride* exudates (without fungus). Gray dots show the fitness of the genes in the rest of the conditions in the fitness browser. The scale shown on both axes are the gene fitness values.


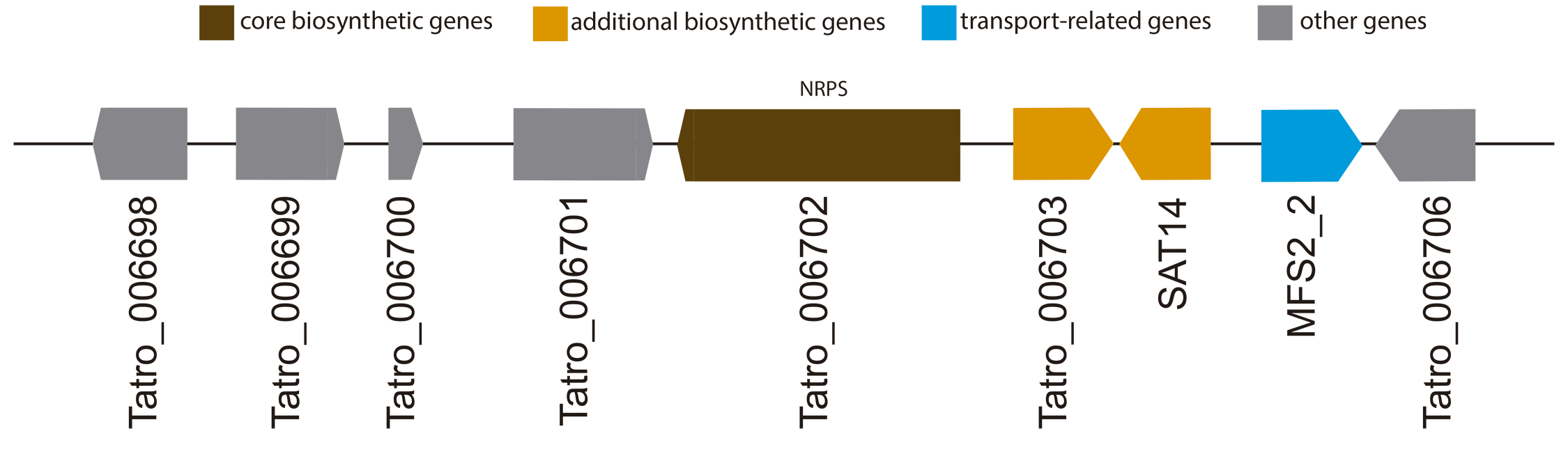


**Fig I. Biosynthetic gene cluster of dimethylcoprogene predicted from the *T. atroviride* genome** (<https://www.ncbi.nlm.nih.gov/assembly/GCA_019297715.1>). This cluster was classified as number 4.1-type non-ribosomal peptide synthetase (NRPS). Dimethylcoprogene may function as a sideophore in *T. atroviride* and has been shown to be produced by other filamentous fungi such as *Alternaria alternata* (7). This BGC shows 100% of similarity to the dimethylcoprogen BCG of *A. alternata.*


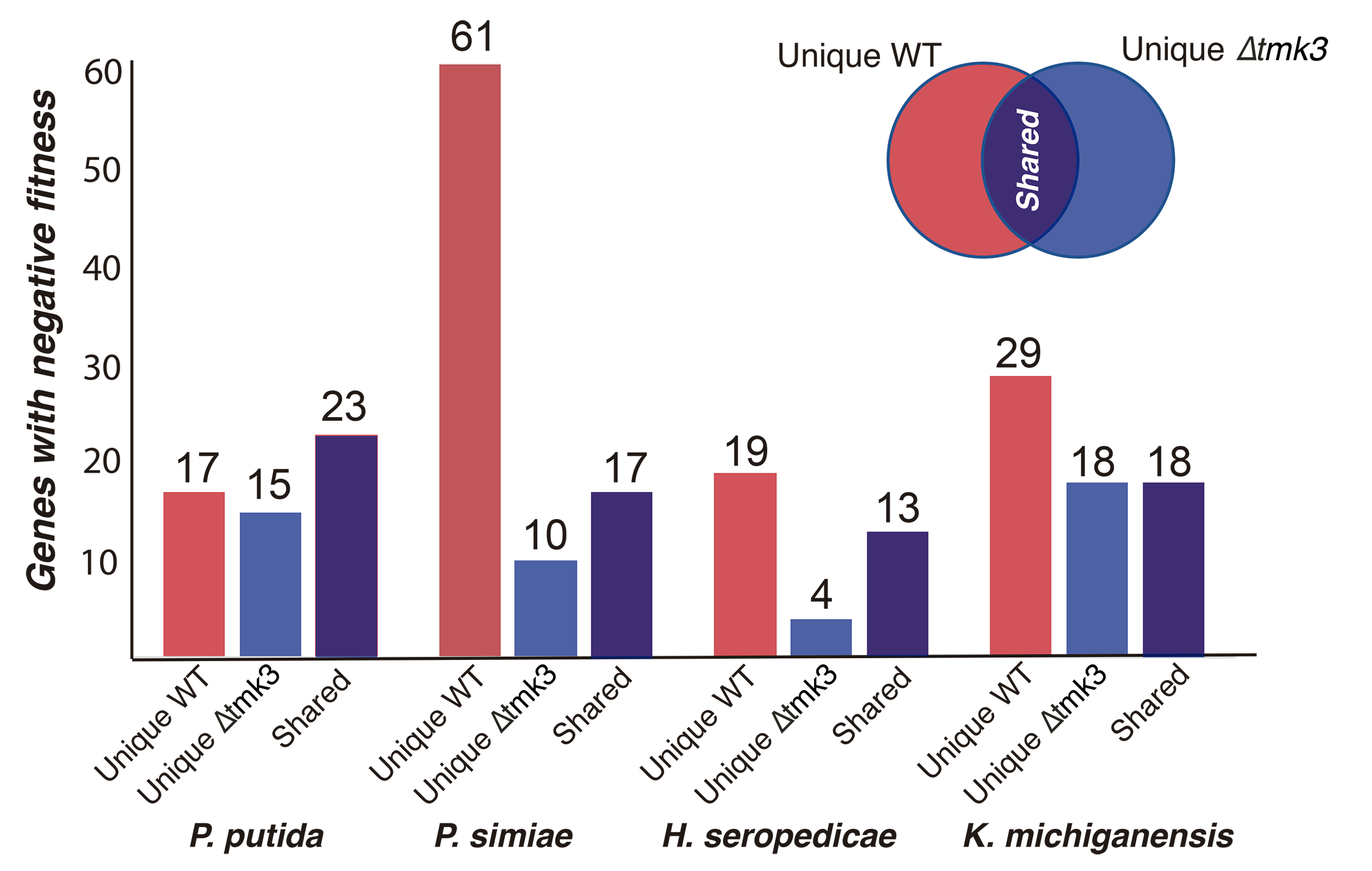


**Fig J.** Number of shared and unique genes important for fitness of the four evaluated bacteria exposed to the exudates of WT *T. atroviride* and the Δ*tmk3* mutant.


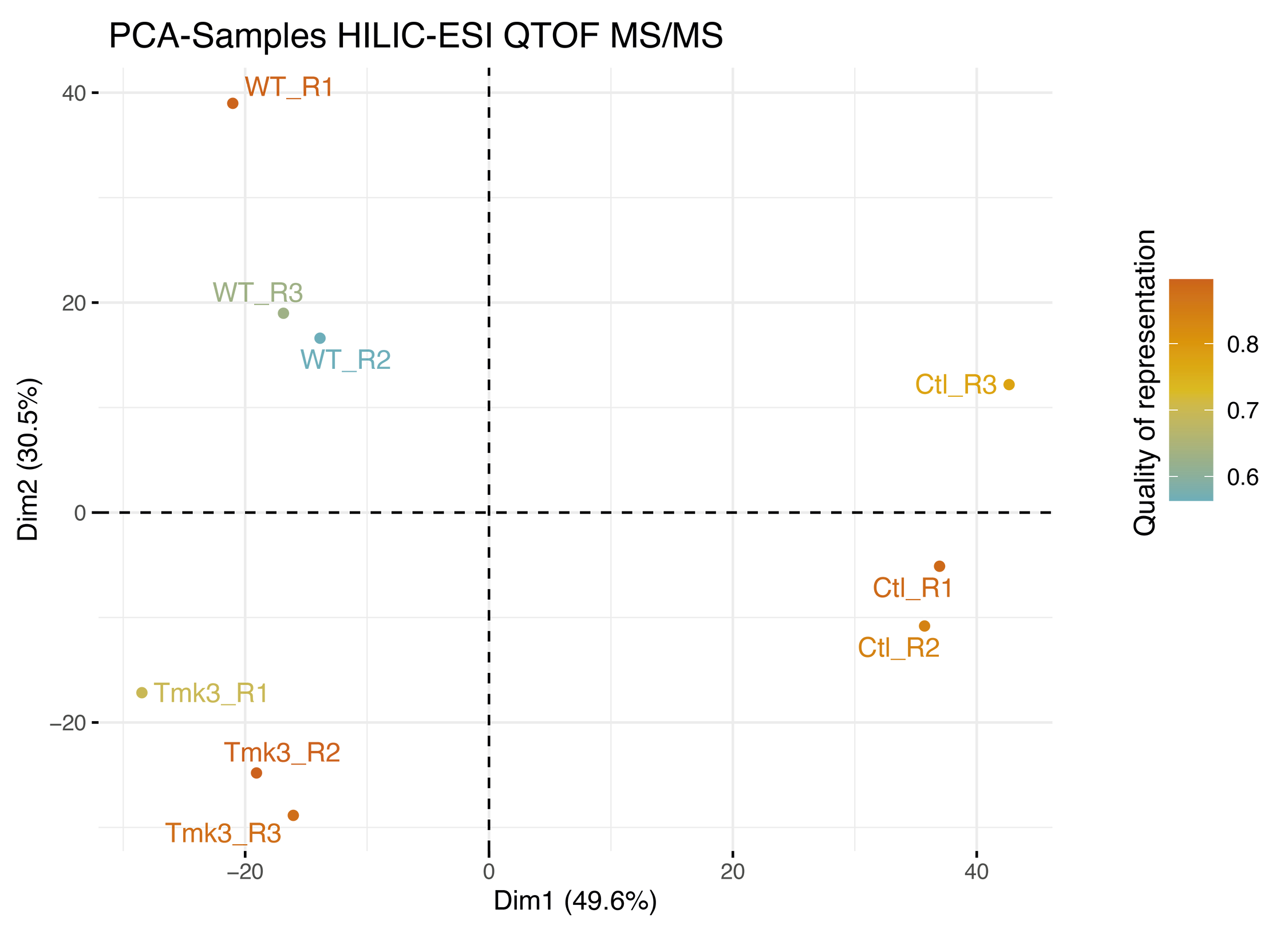


**Fig K.** Principal Component Analysis (PCA) plot of non-targeted metabolomics profile (Dataset 4) showing similarities and differences between the three biological replicates (R1, R2 and R3) of the *T. atroviride* strain (WT), the *Δtmk3* mutant (Tmk3) and the media control (Ctl). The quality of the representation is the proportion of the total variance of the data that is explained by each of the principal components in the scatterplot. Dimension 1 of the PCA represents 49.6% of the variance in the data, while dimension 2 represents 30.5%.

**Supplemental References**

1. Skiada A, Markogiannakis A, Plachouras D, Daikos GL. Adaptive resistance to cationic compounds in *Pseudomonas aeruginosa*. Int J Antimicrob Agents. 2011;37(3):187-93.

2. Gallardo A, Iglesias MR, Ugarte-Ruiz M, Hernandez M, Miguela-Villoldo P, Gutierrez G, et al. Plasmid-mediated Kluyvera-like *arnBCADTEF* operon confers colistin (hetero)resistance to *Escherichia coli.* Antimicrob Agents Chemother. 2023;65(5).

3. Cole BJ, Feltcher ME, Waters RJ, Wetmore KM, Mucyn TS, Ryan EM, et al. Genome-wide identification of bacterial plant colonization genes. PLoS Biol. 2017;15(9):e2002860.

4. Rand JM, Pisithkul T, Clark RL, Thiede JM, Mehrer CR, Agnew DE, et al. A metabolic pathway for catabolizing levulinic acid in bacteria. Nat Microbiol. 2017;2(12):1624-34.

5. Reasoner DJ, Geldreich EE. A new medium for the enumeration and subculture of bacteria from potable water. Appl Environ Microbiol. 1985;49(1):1-7.

6. Brown DW, Lee SH, Kim LH, Ryu JG, Lee S, Seo Y, et al. Identification of a 12-gene fusaric acid biosynthetic gene cluster in Fusarium species through comparative and functional genomics. Mol Plant Microbe Interact. 2015;28(3):319-32.

7. Voss B, Kirschhofer F, Brenner-Weiss G, Fischer R. *Alternaria alternata* uses two siderophore systems for iron acquisition. Sci Rep. 2020;10(1):3587.
